# Mobility of antimicrobial resistance across serovars and disease presentations in non-typhoidal *Salmonella* from animals and humans in Vietnam

**DOI:** 10.1099/mgen.0.000798

**Published:** 2022-05-05

**Authors:** Samuel Bloomfield, Vu Thuy Duong, Ha Thanh Tuyen, James I. Campbell, Nicholas R. Thomson, Julian Parkhill, Hoang Le Phuc, Tran Thi Hong Chau, Duncan J. Maskell, Gabriel G. Perron, Nguyen Minh Ngoc, Lu Lan Vi, Evelien M. Adriaenssens, Stephen Baker, Alison E. Mather

**Affiliations:** ^1^​ Quadram Institute Bioscience, Norwich Research Park, Norwich, UK; ^2^​ Children’s Hospital 1, Ho Chi Minh City, Vietnam; ^3^​ The Hospital for Tropical Diseases, Wellcome Trust Major Overseas Programme, Oxford University Clinical Research Unit, Ho Chi Minh City, Vietnam; ^4^​ Wellcome Sanger Institute, Wellcome Genome Campus, Hinxton, UK; ^5^​ Department of Veterinary Medicine, University of Cambridge, Cambridge, UK; ^6^​ University of Melbourne, Melbourne, Australia; ^7^​ Department of Biology, Bard College, Annandale-on-Hudson, New York, USA; ^8^​ Children’s Hospital 2, Ho Chi Minh City, Vietnam; ^9^​ Department of Medicine, University of Cambridge School of Clinical Medicine, Cambridge Biomedical Campus, Cambridge, UK; ^10^​ University of East Anglia, Norwich, UK

**Keywords:** Antimicrobial resistance, chromosome arrangements, insertion sequences, plasmids, *Salmonella*

## Abstract

Non-typhoidal *Salmonella* (NTS) is a major cause of bacterial enterocolitis globally but also causes invasive bloodstream infections. Antimicrobial resistance (AMR) hampers the treatment of these infections and understanding how AMR spreads between NTS may help in developing effective strategies. We investigated NTS isolates associated with invasive disease, diarrhoeal disease and asymptomatic carriage in animals and humans from Vietnam. Isolates included multiple serovars and both common and rare phenotypic AMR profiles; long- and short-read sequencing was used to investigate the genetic mechanisms and genomic backgrounds associated with phenotypic AMR profiles. We demonstrate concordance between most AMR genotypes and phenotypes but identified large genotypic diversity in clinically relevant phenotypes and the high mobility potential of AMR genes (ARGs) in this setting. We found that 84 % of ARGs identified were located on plasmids, most commonly those containing IncHI1A_1 and IncHI1B(R27)_1_R27 replicons (33%), and those containing IncHI2_1 and IncHI2A_1 replicons (31%). The vast majority (95%) of ARGS were found within 10 kbp of IS6/IS26 elements, which provide plasmids with a mechanism to exchange ARGs between plasmids and other parts of the genome. Whole genome sequencing with targeted long-read sequencing applied in a One Health context identified a comparatively limited number of insertion sequences and plasmid replicons associated with AMR. Therefore, in the context of NTS from Vietnam and likely for other settings as well, the mechanisms by which ARGs move contribute to a more successful AMR profile than the specific ARGs, facilitating the adaptation of bacteria to different environments or selection pressures.

## Data Summary

The sequence data generated during the current study are available in the European Nucleotide Archive (ENA; https://www.ebi.ac.uk/ena/) under projects PRJEB1397, PRJEB9121 and PRJEB2973 for Illumina, and PRJEB9562 for PacBio. The accession numbers and metadata of each isolate are described in Table S1 (available in the online version of this article).

**Impact Statement**

Non-typhoidal *Salmonella* (NTS) is a major cause of diarrhoea worldwide and can cause invasive infections. Antimicrobial resistance (AMR) hampers the treatment of these infections and understanding how it spreads may help in developing effective strategies to combat it. This study describes NTS isolates from Vietnam using whole genome sequencing and phenotypic testing to determine what contributes to a successful AMR profile. We found concordance between most AMR genotypes and phenotypes but isolates from the same AMR phenotypes often had different AMR genes. Most AMR genes were found on a small number of plasmid types and in the vicinity of insertion sequences that allow AMR genes to be exchanged between plasmids and other parts of the genome. Our study demonstrates that for NTS from Vietnam, a successful AMR profile is defined by the mechanisms available for AMR genes to move rather than the specific AMR genes the bacteria contain.

## Introduction

Non-typhoidal *Salmonella* (NTS) comprise the >2500 *Salmonella enterica* subspecies *enterica* serovars that are not associated with enteric fever [1]. Diseases caused by these bacteria include enterocolitis, focal infections, and systemic infections. NTS are responsible for 62–132 million cases of enterocolitis each year and are generally considered to be foodborne in origin [2], although evidence suggests a more nuanced epidemiology in different contexts [3]. Direct exposure to infected or colonised animals or contaminated environments [4] and human-to-human transmission [5] are potential sources of human infection.

Invasive non-typhoidal *Salmonella* (iNTS) infections are a systemic illness with non-specific symptoms that vary between patients, but often include fever, pneumonia, diarrhoea, splenomegaly and hepatomegaly [6]. In Vietnam, iNTS infections have a mortality rate of 26%, with HIV infection a risk factor for morbidity and mortality [7].

Vietnam is a lower-middle income country (LMIC) in Southeast Asia and ranked 11^th^ in the world for antimicrobial consumption [8]. This high rate of antimicrobial consumption has been attributed to the purchasing of antimicrobial agents without a prescription, forced out-of-pocket medical costs increasing self-medication with antimicrobial agents, and a lack of pharmaceutical and microbiological services in hospitals supporting the appropriate prescription of antimicrobial agents [9]. In 2016, the Ministry of Agriculture and Rural Development of Vietnam initiated legislation to phase out antimicrobial agents in animal feed [10]. Multidrug resistant (MDR) NTS have been isolated from animal (12.5–76.1 % of NTS) and human sources (12.5–50 % of NTS) in Vietnam, but there is variation with different sampling locations, animal species, and the different number and types of antimicrobial agents used to identify MDR phenotypes [11–13]. Previous research on NTS infections from Vietnam suggests that isolates from asymptomatic humans tend to be more susceptible to antimicrobial agents than those from animals or humans with diarrhoea [14]. Tackling AMR in LMICs remains a challenge, as any strategy must consider a high burden of infectious disease, as well as other systematic factors such as poverty, governance and health systems, but all decisions rely on understanding what contributes to the emergence of AMR [15].

Horizontal gene transfer (HGT) is the main mechanism for the spread of AMR in most bacterial species [16]. HGT can occur through the uptake of DNA from the environment, bacteriophages moving DNA between bacteria or direct transfer of DNA between bacteria. Bacteria can also package and transfer DNA to other bacteria using gene transfer agents [17] and membrane vesicles [18], and transposons enable bacteria to transfer DNA within its genome [19]. Different molecular methods can be exploited to identify plasmids and AMR genes (ARGs) in bacteria. Plasmid extraction and PCR can be used to determine the specific ARGs carried by different plasmid types, but will not provide information on the plasmid structure or on genes conferring other traits, such as those involved in virulence, that may also be carried on the same plasmid(s) [20]. In addition, these methods fail to identify all plasmid replicons or genes and only describe those specifically investigated. Short read whole genome sequencing (WGS) allows the identification of ARGs and plasmid replicons in isolates [21, 22], but due to the difficulty of assembling repetitive regions using short reads, it is more challenging to identify the location of ARGs within genomes with this approach. Bioinformatic tools have been developed to predict whether short-read contigs are from plasmids or chromosomes [23, 24], but this analysis can be complicated by plasmid regions that have been inserted into chromosomes and fragmented assemblies where plasmids are comprised of multiple contigs. Long read WGS data, especially when combined with short read WGS data permit complete reconstruction of plasmids and chromosomes, and facilitates the identification and localization of ARGs and the mobile genetic elements with which they are associated [25, 26].

In this study we used a combination of long and short read WGS, along with phenotypic testing, to investigate the mechanisms and genomic backgrounds associated with successful AMR profiles that span NTS serovars, disease presentations and host species in Vietnam.

## Methods

### Antimicrobial susceptibility testing

Antimicrobial susceptibility testing was performed on Mueller-Hinton agar using disc diffusion as recommended by Clinical and Laboratory Standards Institute (CLSI) guidelines [27] against: ampicillin, amoxicillin/clavulanic acid, ceftazidime, ceftriaxone, chloramphenicol, ciprofloxacin, gentamicin, nalidixic acid, and trimethoprim.

### Isolates

Eighty-six isolates of non-typhoidal *Salmonella enterica* subspecies *enterica* were selected from the bacterial collections held at the Oxford University Clinical Research Unit (OUCRU) in Ho Chi Minh City, Vietnam. These were selected on the basis of ‘rare’ and ‘common’ (successful) phenotypic AMR profiles. Rare phenotypic AMR profiles were defined as those found in three or fewer isolates from the OUCRU Vietnam collection and common (successful) AMR profiles as those identified in more than ten isolates of at least three different serovars and with resistance to at least three of the considered drugs. Phenotypic AMR profiles were based on the combination of phenotypic resistance to the antimicrobial drugs examined through susceptibility testing. All isolates with rare profiles were selected for inclusion in this study (*n*=46) and 40 isolates were selected with common phenotypes.

### Sequencing

Short read genome sequence data were generated by extracting genomic DNA using the Wizard genomic DNA purification kit (Promega, USA) and sequenced on an Illumina HiSeq2000 platform (San Diego, CA, USA) as 100 bp paired-ends reads.

Long read genome sequencing data were generated by extracting genomic DNA using phenol-chloroform (as described in Mather *et al.* [22]) and sequencing on the Pacific Bioscience platform with P2-B6 chemistry (one single-molecule, real-time (SMRT) cell per genome).

### Assembly

Trimmomatic v0.36 [28] was used to trim the paired Illumina reads. The PacBio and trimmed Illumina reads for each *Salmonella* isolate were assembled using Unicycler v0.4.8 [29] in ‘bold’ mode and Flye v2.7 [30], followed by five rounds of aligning Illumina reads to the assembly using the Burrows-Wheeler Aligner (BWA) v0.7.17 [31] and correcting mismatches using Pilon v1.22 [32]. Socru v2.2.3 [33] was used to split assembled chromosomes into fragments separated by ribosomal operons and determine the order of these fragments. BWA was used to align the Illumina and PacBio reads to the assemblies to determine read depths, and variants between the assemblies and Illumina reads identified.

As the long read genome sequencing process preferentially selects for longer fragments, to ensure the hybrid assemblies were not missing any small plasmids/ARGs compared to those identified in the short read data, ART v2.5.8 [34] was used to simulate paired Illumina reads from the assemblies. ARIBA v2.14.4 [35] was used to search the Illumina and simulated reads against the ResFinder [36] and PlasmidFinder [37] databases. Abricate v0.9.9 (https://github.com/tseemann/abricate) was used to help investigate any discrepant ARIBA results.

Hybrid assembly quality criteria were as follows:

1. Fewer than 20 variants identified with Illumina reads and fewer than 30 contigs.
2. The Socru results did not identify duplicate or unknown fragments.
3. The Socru arrangements were consistent between all assembly algorithms for the isolate.
4. No missing ARGs in the assembly compared to the Illumina reads.
5. No missing plasmids compared to the Illumina (the exception was small plasmid Col_BS512 that were found in some Illumina datasets but were missing from all assemblies).

For assemblies that passed QC, the assembly with the fewest variants and highest number of circular contigs was chosen for further analysis.

### Serovars

SISTR v0.3.1 [38] was performed on assembled genomes as a secondary check on serovar classification; in the case of mismatches, the SISTR results were used.

### Genes of interest

Abricate with the ResFinder, PlasmidFinder and Virulence Finder databases (VFDB) [39] were used to identify AMR determinants and plasmid replicons, and the genomic location (chromosome or plasmid) of ARGs. Abricate matches with identity and coverage values over 95 % were accepted. Some plasmid sequences contained multiple plasmid replicons; when two plasmid replicons were always found in the same contigs for all genomes investigated they were regarded as one plasmid type (e.g., IncHI2_1 and IncHI2A_1 became IncHI2_1/IncHI2A_1).

The BacMet [40] database consists of the amino acid sequences of proteins associated with resistance to heavy metals and antibacterial biocides. tBLASTn v2.9.0 [41] was used with the BacMet amino acid sequences to interrogate the assemblies; matches with identity and coverage values over 95 % were accepted.

Fluoroquinolone-associated mutations were identified using a database formed from the *gyrA*, *gyrB*, *parC* and *parE* genes of *Salmonella enterica* serovar Typhimurium LT2 (NC_003197.2). ARIBA was used to align reads against these genes and identify mutations in these genes associated with quinolone resistance.

### AMR phenotype-genotype

For each antimicrobial class tested, phenotypic resistance was classified as intermediate or resistant according to CLSI guidelines to at least one of the antimicrobials in the class tested, and genotypic evidence was classified as the presence of at least one of the genes in the ResFinder databases and, for fluoroquinolones, the presence of a mutation in the quinolone resistance-determining region (QRDR) of the *gyrA, gyrB*, *parC* and *parE* genes were also regarded as genotypic evidence [42]. The presence of the cryptic aminoglycoside resistance gene *aadA* was not regarded as evidence of aminoglycoside resistance [43].

For each phenotype, the genetic elements predicted to be responsible for the phenotype were extracted and compared. Some phenotypes consisted of one isolate after QC and were excluded from phenotype comparisons.

### Maximum likelihood phylogenetic tree

The *Salmonella* assemblies were annotated using Prokka [44] and clustered using Roary v3.11.2 [45] with 95 % identity and coverage cut-offs. MAFFT v7.475 [46] was used to form a core gene alignment and RaxML v8.0.0 [47] was used to create a maximum likelihood tree from this core gene alignment using a Generalized Time Reversible model [48]. This process was repeated with the genome of the *Escherichia coli* genome (NC_000913.3) to root the tree. Evolview v3 [49] was used to annotate metadata and plasmid types to the maximum likelihood tree.

### Prophage analysis

Prophage sequences and associated genes were identified within the assemblies using PHASTER [50]. vContact2 v0.9.15 [51] was used to cluster the prophage sequences into groups. Prophage sequences were regarded as intact if they contained phage structural genes (capsid, coat, envelope, fibre, head, injection, plate, portal, tail or virion genes) and a phage integrase or recombinase gene. Genes of interest (AMR, metal-tolerance and virulence genes) were regarded as associated with a prophage sequence if they were located between prophage genes in the assemblies.

### Insertion sequences

Insertion sequences (IS) were identified within the assemblies using ISEScan v1.7.1 [52]. ISs that were identified as ‘complete’ were further analysed. To evaluate potential mobility, the number of AMR, virulence and metal tolerance genes within 10 kb of an IS was determined. The same analysis was also conducted for all genomic distances between 0–10 kilobases (kb) to evaluate the choice of 10 kb as a cut-off.

### Plasmid alignments

Plasmid contigs were annotated using Prokka and clustered using Roary as previously mentioned. The gene presence-absence dendrogram produced was used to determine which plasmid sequences were closely related. For plasmid sequences that appeared to be inserted into the chromosome, BLASTn v2.9.0 [53] was used to determine where the plasmid sequence started and ended in the chromosome, with the other plasmid type sequences as the query and the inserted plasmid as the subject. MOB typer v1.4.9 [54] was used to determine the mobility of each plasmid sequence. Evolview [49] was used to annotate metadata on to the dendrograms. EasyFig v2.2.4 [55] was used to align the plasmid sequences and highlight genes of interest and insertion sequences contained within the plasmids. BLASTn was used to determine the query coverage amongst sequences belonging to each plasmid type.

Linear regression was used to model the total number of AMR, virulence and metal-tolerance genes with the isolate metadata, plasmid type and the number of each IS type on the plasmid sequences. Partial-F tests were used to determine if the potential explanatory variables significantly improved the fit of the model.

## Results

### Assembly quality control and phylogenetics

**Fig. 1.**
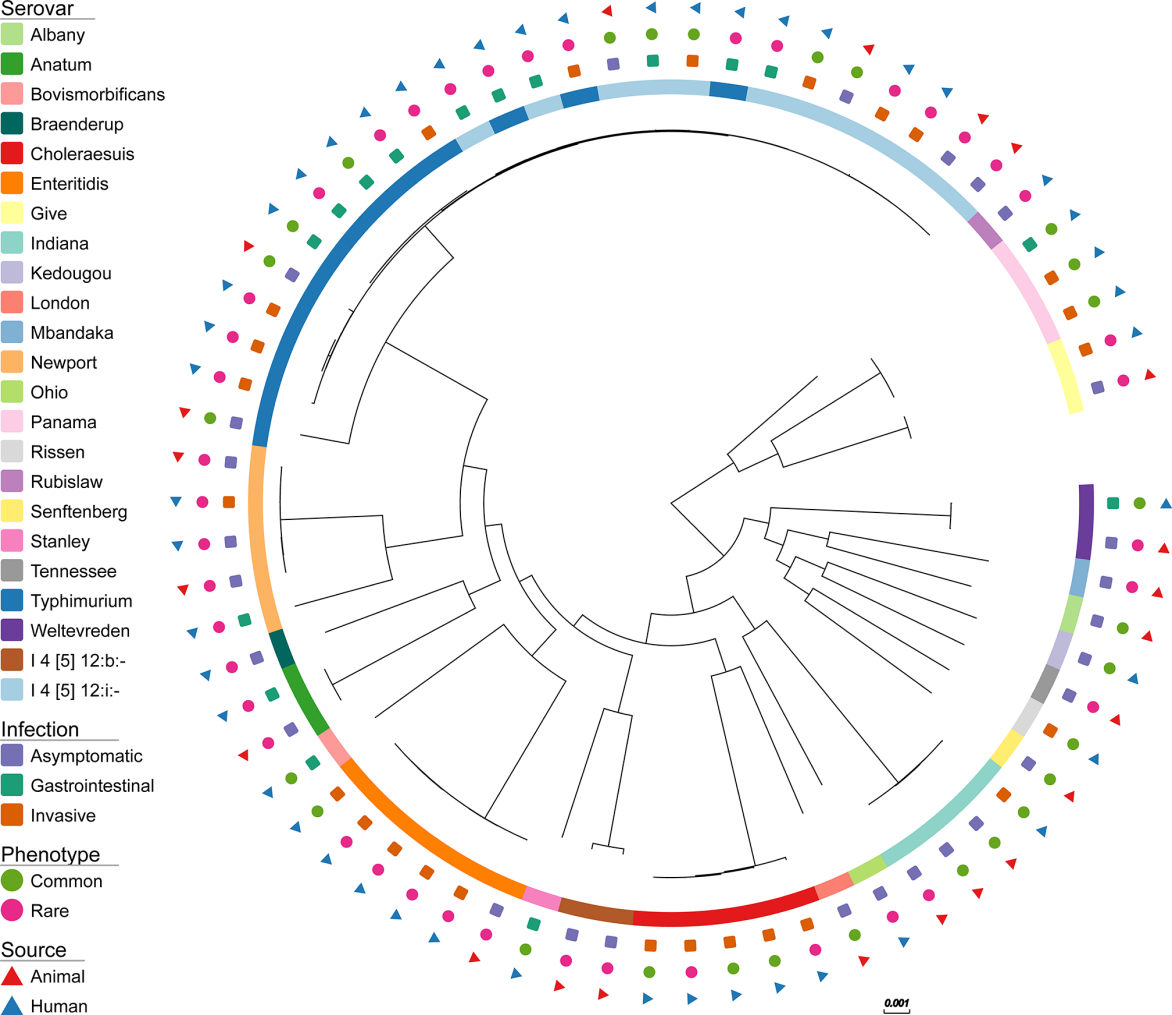
Maximum likelihood tree of 68 non-typhoidal *Salmonella* isolates. Coloured symbols represent serovars (inner ring), type of infection (second ring), phenotypic AMR profile commonality (third ring) and source (outer ring). The phylogenetic branch lengths are given in nucleotide substitutions per site, therefore a branch of length 0.001 (as represented by the scale bar) equates to 3179 substitutions, given that a 3178949 bp core gene alignment was used to estimate it; tree was rooted using *Escherichia coli* (NC_000913.3).

Illumina and PacBio WGS were performed in tandem on 86 NTS isolates from Vietnam; 68 had assemblies that met the defined quality control criteria and therefore further analysed. The 68 *Salmonella* isolates formed serovar-associated clades (Fig. 1), with *S.* Typhimurium and I 4 [5], 12:i:- (monophasic variant of *S.* Typhimurium [56]) isolates found in the same clade.

### Phenotypic-genotypic concordant evidence of AMR

Antimicrobial susceptibility testing was performed to reconcile these data with the presence of genetic elements known to confer AMR. Genomic analysis identified 49 different putative ARGs and five mutations in the QRDR of *gyrA* (D87G, D87N, D87Y, S83F and S83Y) amongst the 68 NTS isolates. For most isolates, there was good concordance between AMR phenotype and genotype (Appendix A). Phenotypic antimicrobial susceptibility testing determined that the 68 NTS isolates fell into phenotypic AMR profiles based on resistance to five antimicrobial classes: aminoglycoside (Am), beta-lactam (Bl), chloramphenicol (Ch), fluoroquinolone (Fl) and trimethoprim (Tr). Of these phenotypic AMR profiles, twelve consisted of more than one isolate; we compared predicted genetic elements responsible for these phenotypes. Only one of the twelve phenotypic AMR profiles (ChFlTr) consisted of isolates that all contained the same genetic causes. All other phenotypic profiles were represented by diverse genotypic AMR determinants.

### Genomic location of AMR, virulence and metal-tolerance genes

ARGs have been found co-located with metal-tolerance genes and therefore exposure to heavy metals can co-select for AMR [57]. ARGs have also been found on the same plasmids as virulence genes [58], so we also investigated metal-tolerance determinants and virulence genes. The proportion of each gene group was similar amongst serovars (Appendix B), apart from the *Salmonella* serovars Ohio (*n*=1), Rubislaw (*n*=1), Tennessee (*n*=1), Weltevreden (*n*=2) and I 4 [5], 12:b:- (*n*=2) in which no ARGs were identified, but this may be due to a small number of isolates investigated belonging to these serovars. Overall, 84 % of identified ARGs were encoded on plasmid replicon-containing contigs, compared to 1 % of virulence and 7 % of metal-tolerance genes. The majority of ARGs were located on contigs containing IncHI1A_1/IncHI1B(R27)_1_R27 (33%) and IncHI2_1/IncHI2A_1 (31%) replicon types. Plasmid contig groupings were complicated by those encoding multiple replicons, e.g., all contigs with the IncFIA(HI1)_1_HI1 replicon also had the IncHI1A_1/IncHI1B(R27)_1_R27 replicon, but not vice versa.

Pan-genome analysis revealed that no ARGs were incorporated in the core genome (defined as presence in >95 % of isolates), compared to 65 % of virulence and 60 % of metal-tolerance genes. The metal-tolerance genes encoded resistance to 47 metals and antibacterial biocides; the NTS isolates investigated contained one or more core resistance genes associated with 36 of these compounds.

Insertion sequences (*n*=1982) were found throughout the plasmid (*n*=678) and chromosomal (*n*=1304) sequences of the *Salmonella* genomes. In total, 98 % of ARGs were located within 10 kb of insertion sequences, compared to 7 % of virulence and 5 % of metal-tolerance genes. However, this insertion sequence analysis was complicated by some genes being in the vicinity of multiple insertion sequence types. Regardless of the genomic distance used as a cut-off (Appendix C) we confirmed that a larger proportion of ARGs were found in proximity to insertion sequences compared to virulence or metal-tolerance genes. IS6/IS26 comprised 21 % of all identified insertion sequences, but 95 % of ARGs were within 10 kb of this insertion sequence. Six ARG types were identified that were never associated with IS6/IS26, and five plasmid replicons were identified that were never found on the same contigs as IS6/IS26-associated ARGs. However, few copies of these ARGs (1-3) and plasmid replicons (1-5) were identified amongst the 68 NTS isolates investigated, preventing comparisons between specific ARGs, location and dissociation with IS6/IS26.

Prophage analysis identified 551 different prophage fragments in the 68 NTS genomes. Of these, 162 (29.4 %) fragments were predicted to be intact based on their gene complement and 86 fragments (15.6%) were located on plasmids. A significantly smaller proportion of prophage sequences on plasmids were predicted to be intact (19.7%) compared to those located chromosomally (31.1%) (Fisher’s Exact test; *P*=0.04). However, two circular plasmids were identified that almost entirely (>99 %) consisted of PHASTER-predicted phage genome and both contained the pO111 plasmid replicon, making them phage-like plasmids. BLASTn found 79–80 % coverage between these plasmids and a phage P1 representative (NC_005856.1) [59]. AMR, virulence and metal-tolerance genes were found in regions defined as prophages by PHASTER, but upon further investigation either 1) the prophage sequences were not considered intact or 2) if the prophage sequences were considered intact, the resistance or virulence genes of interest were adjacent to the prophage genes and not incorporated within the prophage sequence. A small proportion of insertion sequences (1.7%) were found amongst phage genes in intact phage genomes, but the proportions differed amongst different insertion sequence types (Appendix D).

### Plasmid alignments

**Fig. 2.**
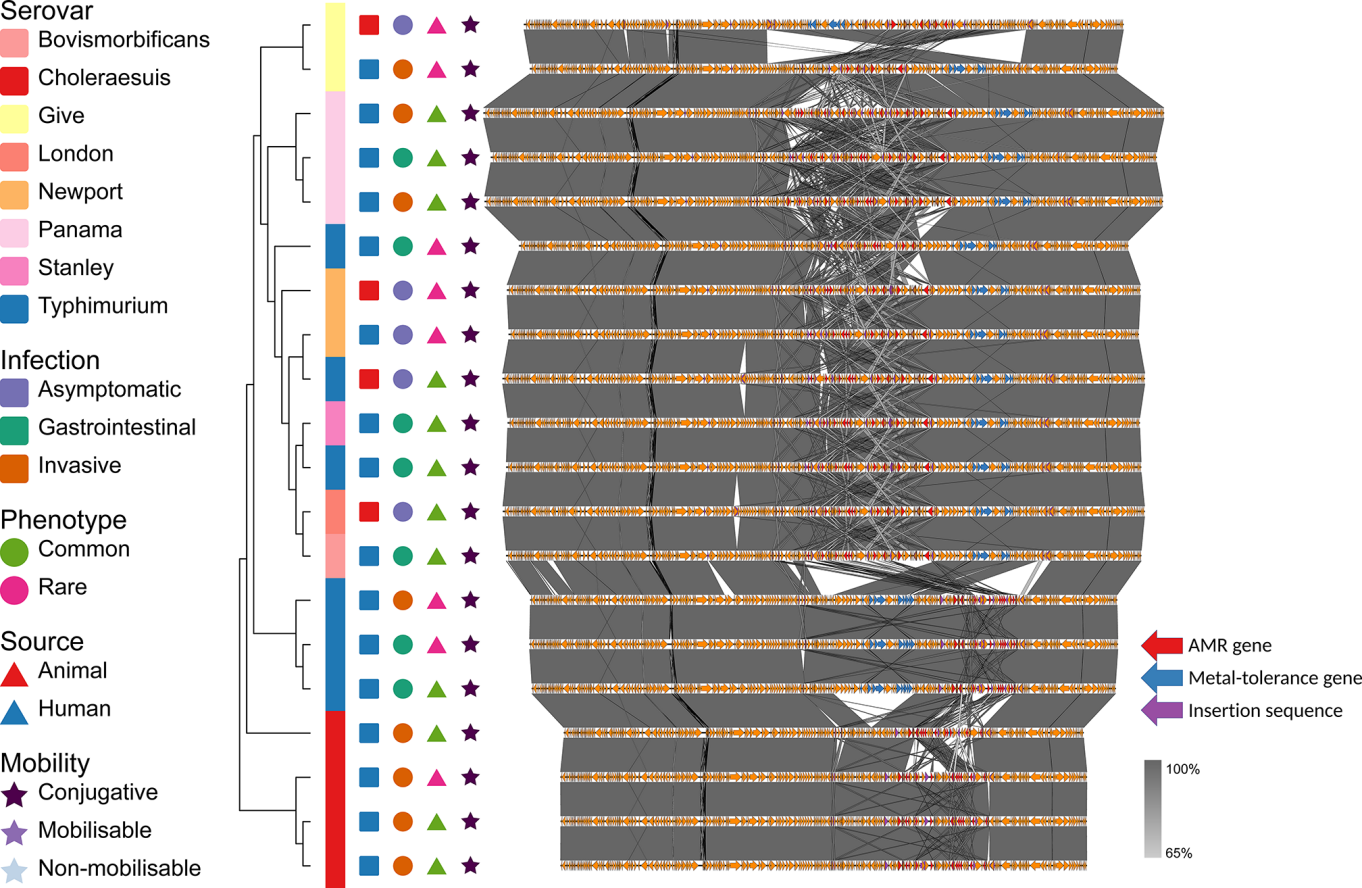
IncHI1A_1/IncHI1B(R27)_1_R27 plasmid alignment. Dendrogram based on gene presence-absence; symbols representing serovar, type of infection, phenotypic AMR profile commonality, source and plasmid mobility; and alignment of plasmid contigs. Arrows represent genes: red arrows represent ARGs, blue arrows represent metal-tolerance genes, and purple arrows represent ISs. Bars between contigs represent BLASTn alignments.

**Fig. 3.**
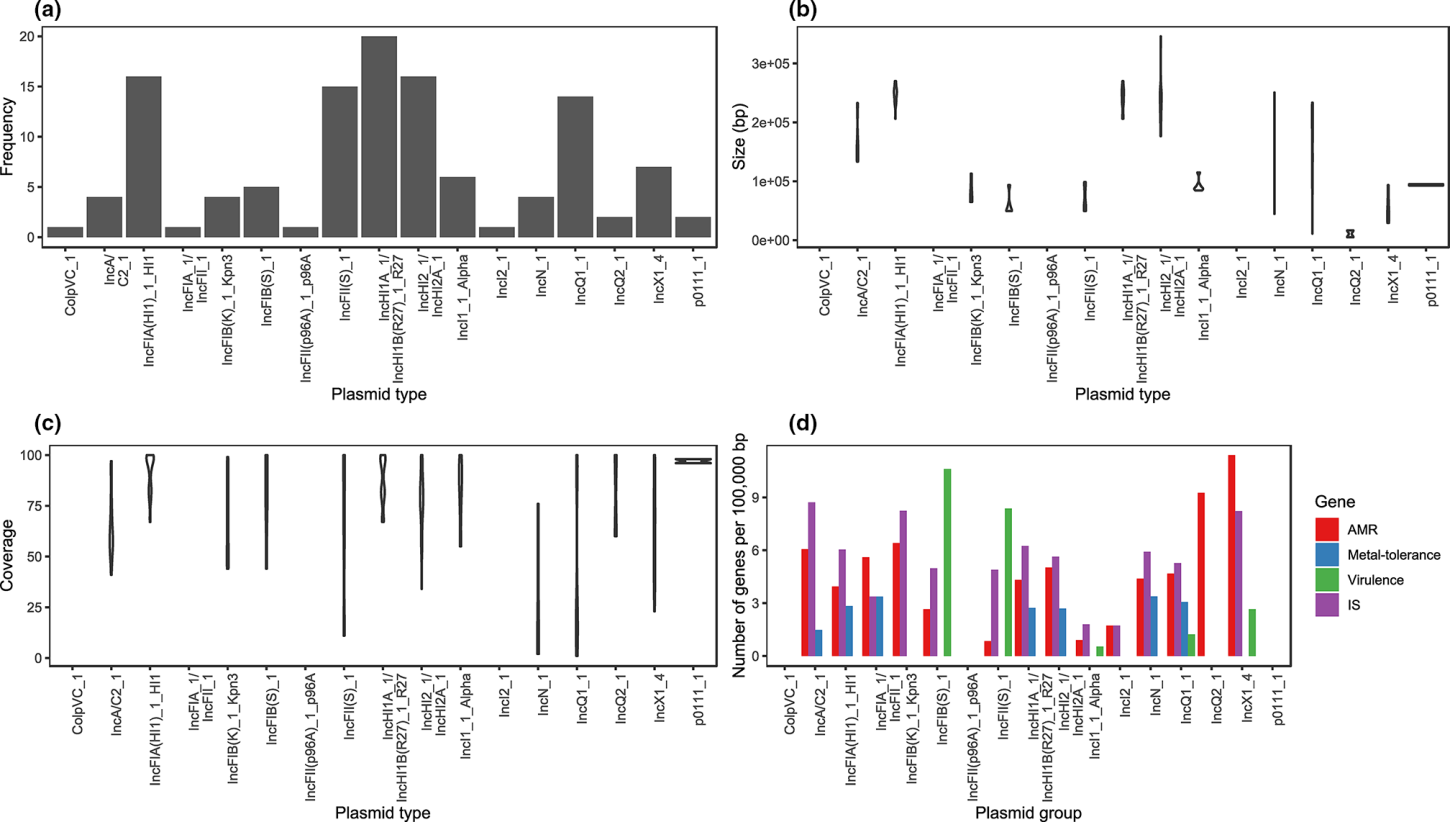
Plasmid content comparison. (**a**) Bar graph of the number of each plasmid type identified, (**b**) violin plot of the size of each sequence belonging to each plasmid type, (**c**) violin plot of the percentage coverage between sequence for each plasmid type, and (**d**) the number of AMR genes (red), virulence genes (green), metal-tolerance genes (blue) and insertion sequences (purple) on plasmids from each plasmid group.

Plasmid analysis identified 87 contigs with plasmid replicons from 62 of the 68 NTS isolates investigated. Of these, 66 (76 %) contained ARGs, 44 (51 %) contained metal-tolerance genes, and 20 (23 %) contained virulence genes. These contigs contained 15 unique replicons with some encompassing multiple replicons. Notably, the IncHI1A_1/IncHI1B(R27)_1_R27 plasmid type was associated with the greatest number of ARGs (Fig. 2), although this plasmid did not have the highest density of ARGs (Fig. 3). This plasmid type contained a conserved backbone, and a variable region with a high degree of potential gene movement, including ARGs. The IncFIB(K)_1_Kpn3 and IncA_C2_1 plasmid types also had conserved backbones and variable regions with high AMR diversity, but were not common (Appendix E). IncHI2_1/IncHI2A_1, IncQ1_1 and IncX1 plasmid types consisted of sequences with limited sequence similarity, but all contained ARGs and some were inserted into the chromosome. The IncFII(S)_1, IncI1_1_Alpha and IncN_1 plasmid types consisted of sequences with and without ARGs. Plasmid types IncHI1A_1/IncHI1B(R27)_1_R27, IncFIB(K)_1_Kpn3, IncA_C2_1, IncHI2_1/IncHI2A_1, IncQ1_1 and IncX1 were found in isolates from various serovars, sources, infections and phenotype; ARGs in these plasmids tended to be located in variable regions with many nearby insertion sequences. For the colpVC_1, IncFII(p96A)_1_p96A, IncFIA_1/IncFII_1, IncI2_1, IncQ2 and pO111_1 plasmid types, only one or two sequences were identified and analysed, precluding any generalisations of their gene content.

Plasmids belonging to each plasmid type varied in their range of lengths, with some length ranges <50 kbp (e.g., IncI1_1_Alpha) and some >200 kbp (e.g., IncN_1). Different plasmids also varied in their degree of sequence identity, with some sharing >65 % of total sequence identity (e.g., IncHI1A_1/IncHI1B(R27)_1_R27) and some showing <10 % of total sequence identity (e.g. IncN_1). The frequency of the genes of interest (i.e. AMR, metal-tolerance and virulence genes) and insertion sequences also varied between plasmid types.

Linear regression analysis demonstrated that the number of IS6/IS26 insertion sequences was significantly associated with the number of ARGs (*P*=0.002). Virulence genes were found to be associated with plasmid type (*P*=0.0002); in particular the number of virulence genes was positively associated with IncFII(S)_1 (*P*=0.01) and negatively associated with IncHI1A_1/IncHI1B(R27)_1_R27 (*P*=0.03). The number of IS1 insertion sequences were also significantly associated with the number of virulence genes (*P*=0.007). Metal-tolerance genes were associated with plasmid type (*P*=0.001), with the number of metal-tolerance genes positively associated with IncFIA(HI1)_1_HI1 (*P*=0.006), IncHI2_1/IncHI2A_1 (*P*=0.006), IncN_1 (*P*=0.04) and IncQ1_1 (*P*=0.01). The number of IS1182 (*P*=0.005) and ISL3 (*P*=0.003) insertion sequences were also positively associated with the number of metal-tolerance genes. However, plasmids with no ARGs, virulence or metal-tolerance genes, led to relatively poor model fit.

### Invasive *Salmonella* genomic associations

**Fig. 4.**
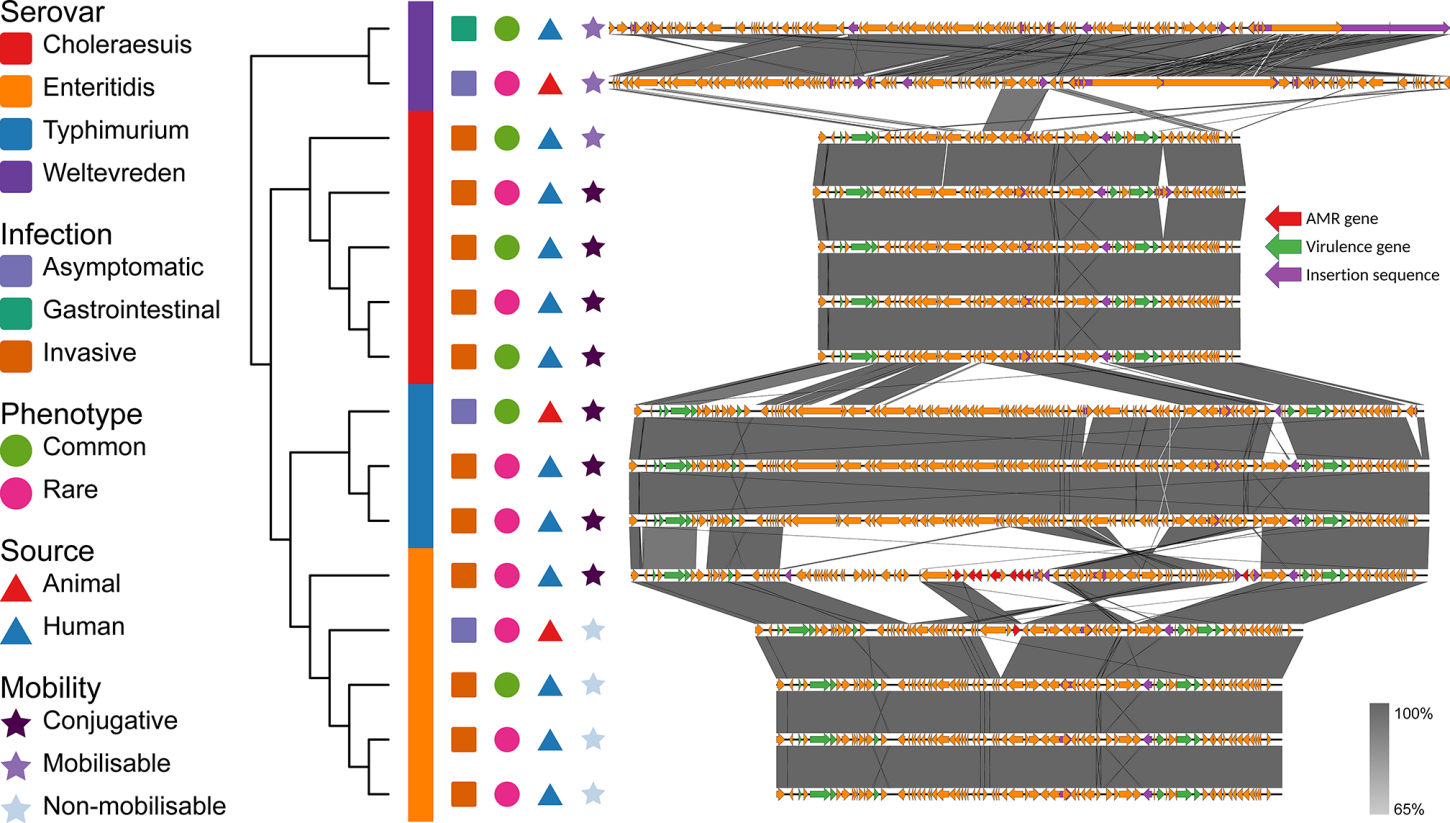
IncFII(S)_1 plasmid alignment. Dendrogram based on gene presence-absence; symbols representing serovar, type of infection, phenotypic AMR profile commonality, source and plasmid mobility; and alignment of plasmid contigs. Arrows represent genes: red arrows represent ARGs, green arrows represent virulence genes and purple arrows represent ISs. Bars between contigs represent BLASTn alignments.

The *Salmonella* virulence plasmid (SVP; IncFII(S)_1 plasmid type) was found in four serovars (Fig. 4). The SVP in the Choleraesuis, Typhimurium and Enteritidis sequences all contained virulence genes encoding the type III secretion system effectors, *spvB* and *spvC*, the spv operon regulator, *spvR,* and the plasmid-associated fimbriae genes, *pefA*, *pefB*, *pefC* and *pefD.* Most also contained *rck,* which encodes an outer membrane protein involved in serum resistance. The two sequences from the Weltevreden serovar lacked any virulence genes and were not isolated from invasive infections. Significantly, 11 out of 25 (44 %) invasive isolates contained an SVP, compared to one out of 16 gastrointestinal isolates (6.3%), and three out of 27 asymptomatic isolates (11.1%) (*P*=0.0005, Fisher’s Exact test). As a note, the isolates collected from asymptomatic infections with an SVP plasmid were sampled from animals. Three genomes had uncommon chromosome arrangements and were all from iNTS cases belonging to *S.* Enteritidis (*n*=2) and *S.* Choleraesuis (*n*=1) serovars (Appendix F).

## Discussion

In this study, we evaluated genomic mechanisms and context in rare and common phenotypic AMR profiles found across different NTS serovars, disease presentations and host species. Of the twelve phenotypic AMR profiles that were represented by multiple isolates, only one comprised isolates with identical genetic elements responsible for the phenotype. For the remaining profiles, different combinations of AMR genes and mutations were responsible for the phenotypes, in line with previous comparisons of *Salmonella* AMR genotypes and phenotypes [60]. While phenotypic testing is important from a clinical perspective, only genotypic data provides information on the mechanisms responsible for resistance, and the greater resolution is essential for retrospective analyses on the evolution and spread of AMR.

Comparisons of genotypic and phenotypic AMR data revealed concordance for most of the isolates. Variation in ARG expression may result in non-concordant data [61], suggesting that WGS is effective at tracking AMR, but further work is required to decrease its error rates, especially genes falsely associated with phenotypic aminoglycoside resistance. In addition, novel AMR mechanisms absent from the database could also result in non-concordant data, but newly developed functional metagenomic analysis promises to reveal previously unidentified AMR mechanisms [62]. iNTS infections result from NTS entering the bloodstream, resulting in a systemic illness [6]. In this study, 25 of the 68 NTS isolates were associated with invasive infections. These isolates belonged to nine serovars and SVPs were found in three of these serovars: Choleraesuis (5/5), Enteritidis (4/5) and Typhimurium (2/5). SVPs are found in many *Salmonella* serovars but their role in salmonellosis pathogenesis is not well defined [63]. Some studies have found the SVPs are associated with isolates from invasive infections [64, 65], whilst others have found the opposite [66]. However, these studies solely focused on *Salmonella* Typhimurium. Chiu *et al.* [67] found that NTS serovars primarily causing invasive human infections commonly harboured SVPs, but serovars primarily involved with enterocolitis had similar proportions of isolates from enterocolitis and invasive infections with SVPs. In this study, SVPs were associated with isolates from invasive infections in serovars primarily causing invasive infections (e.g., Choleraesuis) and for those primarily causing enterocolitis (e.g., Typhimurium), but no SVPs were identified in 6/9 serovars isolated during invasive infections. In addition, variation in the gene content of the SVPs from different serovars was observed. Most of the studies regarding SVP prevalence have used PCR-based methods for detection, but to determine how SVPs relate to salmonellosis pathogenesis, long-read sequencing is required to determine the entire gene content of the plasmids and how this relates to isolates from different serovars and infections.

NTS chromosomes contain seven ribosomal operons, dividing the chromosome into seven fragments. Due to the large amount of sequence similarity between the operons, homologous recombination can occur between them, rearranging the fragments. Page *et al.* [33] found that most *Salmonella enterica* isolates have the same chromosome arrangement (GS1.0), but 28 other arrangements have been described. In this study, three non-GS1.0 chromosome arrangements were identified; all were found in isolates from invasive infections. Chromosome rearrangements have been previously identified in both host-specific *Salmonella* serovars [68] and *S. enterica* serovar Typhi causing carriage [69]. While further research is required to determine the impact of different chromosomal structures on iNTS infections, the current study suggests a link between iNTS infections and chromosomal rearrangements in *Salmonella *.

The role of prophages in AMR transmission is not well understood. Phages have been shown to be able to transfer ARGs between *Salmonella in vitro* [70], but few phage genomes carry ARGs [71]. While we identified many prophage sequences in the genomes of the NTS analysed, no intact prophage sequences were identified with ARGs integrated in the prophage genome itself, i.e. between the phage genes. It is, therefore, most likely that prophage-mediated transfer of ARG is through specialised transduction in which random parts of the bacterial genome get packaged into the phage capsids and spread [21]. A third potential way of dissemination identified here, could be through phage-like plasmid mobilisation of ARGs [59]. However, the phage-like plasmids identified in this study did not carry any ARGs. A small proportion of the insertion sequences were also found in intact prophage genomes, suggesting that phages may introduce insertion sequences to NTS genomes, providing an extra level of mobility for gene movement.

IS6/IS26 are a family of ISs [72] that have been shown to help disseminate ARGs in multiple bacterial species [73, 74]. IS6/IS26 involvement in AMR movement is explained by its two modes of transport: replicative transposition and a conservative process. Replicative transposition occurs by the insertion sequence replicating itself and integrating the copy into another region [75], explaining how multiple copies of IS6/IS26 were found in the same plasmid sequences. Multiple copies can also be accumulated if the donor DNA is transiently present in the cell. The conservative process involves a pair of IS6/IS26 and the region between them (i.e. a transposon) inserting into another region, preferentially next to another IS6/IS26 [76], without replication, allowing plasmids containing IS6/IS26 to exchange ARGs. The close proximity of IS6/IS26 genes to ARGs and positive correlation between the number of these ISs and ARGs on plasmids, supports previous work showing that IS6/IS26 contributes to the movement of ARGs between different genomic regions [77].

In this study, the context of ARGs was compared to virulence and metal-tolerance genes, as all gene categories can move via horizontal gene transfer and can provide an evolutionary advantage to the bacterium [78]. We found that ARGs were present more frequently on plasmids and other mobile genetic elements as opposed to located on the chromosomes. NTS have accumulated ARGs under varying selective pressures following the introduction of antimicrobials for prophylaxis, to treat infections and to improve agricultural yields [79, 80]. While these ARGs can have a fitness cost on the bacterium, the cost tends to be less for the acquisition of plasmid-borne ARGs than chromosomal mutations and can be more readily lost in the absence of selective pressure [81]. Furthermore, the location of most of the ARGs on plasmids in association with insertion sequences creates a pool of ARGs that can easily be shared between bacteria. However, plasmids often confer a fitness cost on bacteria, including the IncH plasmids that contained most of the AMR genes identified in this study [82]. These costs can be ameliorated through compensatory mutations [83]; transforming diverse *Salmonella* with the plasmids described in this study and conducting fitness assays would provide insight into any potential fitness costs associated with these plasmids.

Compared to AMR and virulence genes, less is known about the history of metal tolerance and antibacterial biocide resistance genes. In this study, these genes were found primarily in chromosomes and not associated with insertion sequences, like virulence genes. However, I 4 [5], 12:i:- has acquired resistance to metals on *Salmonella* Genomic Islands to facilitate survival on livestock feed to which copper and zinc are often added [84, 85]. In addition, Wilson *et al.* [86] found varying minimum inhibitory concentrations (MIC) of NTS to different antibacterial biocides and metals between isolates collected from the same and different serovars, indicating that some environments that NTS colonise select for metal resistance. Further analysis on the acquisition of metal-tolerance genes by NTS, their fitness cost and comparisons with phenotypic data is required to understand the role of these genes in the evolution of NTS.

An aim of this study was to determine the contributing factors to a successful AMR profile, where success is defined as presence in multiple serovars, host species and disease presentations. Initially, successful AMR profiles were defined by phenotypic testing, with rare phenotypic AMR profiles defined as those found in three or fewer isolates from the OUCRU Vietnam collection and common (successful) AMR profiles in more than ten isolates of at least three different serovars and with resistance to at least three of the considered drugs. This study demonstrates that while some AMR profiles are common based on phenotype data, diverse underlying genetic mechanisms contribute to the phenotype. Therefore, at the resolution of genotype there are few common AMR profiles. In addition, long-read sequencing revealed the genomic context of the ARGs, showing that most ARGs were found on plasmids in association with IS6/IS26. This facilitates the movement of ARGs between plasmids and results in a large diversity of ARG profiles within plasmids. Therefore, in the context of NTS from Vietnam and likely for other settings as well, the mechanisms by which ARGs move between NTS contribute more to a successful AMR profile than the specific ARGs, allowing NTS to gain and lose ARGs in response to environments with different antimicrobial agents or other selection pressures.

Long-read sequencing has facilitated ascertainment of the genomic location of ARGs, and therefore the evolutionary pathways of resistant organisms and likely routes of ARG movement. For example, it has been used on carbapenem-resistant *Klebsiella pneumoniae* to identify the plasmids with which carbapenemase genes are associated [87], and the transmission of a single carbapenemase gene from *Pseudomonas aeruginosa* to multiple species of *Citrobacter* via a plasmid [88]. Long-read sequencing has also allowed identification of the mechanisms of emergence of new antimicrobial resistant strains of bacteria, such as *Campylobacter jejuni* ST6964 in New Zealand, where a tetracycline resistance gene moved from a plasmid into the chromosome [25]. As with these studies in organisms other than *Salmonella *, here we have demonstrated the importance of plasmids and insertion sequences, but the mobile genetic elements responsible were not the same in the different bacterial species. Therefore, long-read sequencing of different bacteria from around the world and in many different ecological compartments is required to investigate the predominant mobile genetic elements responsible for AMR transmission.

We have demonstrated the large genotypic diversity underlying clinically relevant phenotypes. The greater resolution offered by identification of specific mechanisms of AMR and the genomic context in which they are found provides insight into the evolution and sources of AMR by identifying the most common vehicles of AMR within their population using an approach which can and has been applied in other settings [89]. Here, the most common vehicles were IncHI1A_1/IncHI1B and IncHI2_1/IncHI2A_1 plasmids and IS6/IS26 elements. Such information can be used in the future to design targeted intervention strategies, such as using CRISPR-Cas systems [90, 91], to have the greatest impact on reducing antimicrobial resistance.

## Supplementary Data

Supplementary material 1
