# Supplementary material for: Mobility of antimicrobial resistance across serovars and disease presentations in non-typhoidal Salmonella from animals and humans in Vietnam
Source: Microb Genom. 2022 May 5;8(5):mgen000798. doi: 10.1099/mgen.0.000798 (PMC9465066; doi:10.1099/mgen.0.000798)
Supplement: Supplementary material 1 [file mgen-8-798-s001.pdf]

Table S1. Isolate metadata

Sample name	Phenotype commonality	Serovar SISTR	Species	Infection	Illumina study accession	Illumina run accession	PacBio study accession	PacBio run accession
2511STDY5908381	Rare	I 4,[5],12:i:-	Chicken	Asymptomatic	PRJEB1397	ERR702346	PRJEB9562	ERR987693
3622STDY6094355	Rare	Indiana	Duck	Asymptomatic	PRJEB9121	ERR1046206	PRJEB9562	ERR987694
3622STDY6094379	Rare	I 4,[5],12:b:-	Chicken	Asymptomatic	PRJEB9121	ERR1046229	PRJEB9562	ERR999933
3622STDY6094415	Rare	Anatum	Chicken	Asymptomatic	PRJEB9121	ERR1046264	PRJEB9562	ERR999934
3622STDY6094208	Rare	Newport	Human	Blood stream infection	PRJEB9121	ERR1069232	PRJEB9562	ERR1007451
68_NTS	Rare	Typhimurium	Human	Blood stream infection	PRJEB2973	ERR161423	PRJEB9562	ERR1007452
148_NTS	Rare	Typhimurium	Human	Blood stream infection	PRJEB2973	ERR161417	PRJEB9562	ERR1007453
151_NTS	Rare	Typhimurium	Human	Blood stream infection	PRJEB2973	ERR161429	PRJEB9562	ERR1016546
3622STDY6094243	Rare	Choleraesuis	Human	Blood stream infection	PRJEB9121	ERR1069309	PRJEB9562	ERR1016547
3622STDY6094277	Rare	Newport	Human	Diarrhea	PRJEB9121	ERR1046133	PRJEB9562	ERR1036247
20081	Rare	I 4,[5],12:i:-	Human	Diarrhea	PRJEB2973	ERR161978	PRJEB9562	ERR1036248
3622STDY6094291	Rare	Anatum	Human	Diarrhea	PRJEB9121	ERR1069236	PRJEB9562	ERR1036249
2511STDY5908405	Rare	I 4,[5],12:i:-	Pig	Asymptomatic	PRJEB1397	ERR702370	PRJEB9562	ERR1100799
3622STDY6094400	Rare	I 4,[5],12:b:-	Chicken	Asymptomatic	PRJEB9121	ERR1046249	PRJEB9562	ERR1100800
3622STDY6094424	Rare	Ohio	Human	Asymptomatic	PRJEB9121	ERR1069240	PRJEB9562	ERR1100802
2511STDY5564064	Rare	Weltevreden	Duck	Asymptomatic	PRJEB1397	ERR387732	PRJEB9562	ERR1109332
596_NTS	Rare	Typhimurium	Human	Blood stream infection	PRJEB2973	ERR161442	PRJEB9562	ERR1109333
3622STDY6094194	Rare	Enteritidis	Human	Blood stream infection	PRJEB9121	ERR1069261	PRJEB9562	ERR1109334
3622STDY6094197	Rare	Choleraesuis	Human	Blood stream infection	PRJEB9121	ERR1069264	PRJEB9562	ERR1109335
3622STDY6094422	Rare	Rubislaw	Human	Asymptomatic	PRJEB9121	ERR1046271	PRJEB9562	ERR1109336
3622STDY6094430	Rare	Give	Chicken	Asymptomatic	PRJEB9121	ERR1046277	PRJEB9562	ERR1109337
3622STDY6452006	Rare	Typhimurium	Human	Diarrhea	PRJEB9121	ERR1788708	PRJEB9562	ERR1109339
20005	Rare	I 4,[5],12:i:-	Human	Diarrhea	PRJEB2973	ERR161401	PRJEB9562	ERR1109340
10045	Rare	Typhimurium	Human	Diarrhea	PRJEB2973	ERR161390	PRJEB9562	ERR1124251
3622STDY6094346	Rare	Newport	Duck	Asymptomatic	PRJEB9121	ERR1046197	PRJEB9562	ERR1223255
3622STDY6094403	Rare	Enteritidis	Chicken	Asymptomatic	PRJEB9121	ERR1046252	PRJEB9562	ERR1223256
3622STDY6094416	Rare	Newport	Human	Asymptomatic	PRJEB9121	ERR1046265	PRJEB9562	ERR1223257
3622STDY6094417	Rare	Tennessee	Chicken	Asymptomatic	PRJEB9121	ERR1046266	PRJEB9562	ERR1223258
3622STDY6094421	Rare	Newport	Chicken	Asymptomatic	PRJEB9121	ERR1046270	PRJEB9562	ERR1223259
3622STDY6094431	Rare	Mbandaka	Chicken	Asymptomatic	PRJEB9121	ERR1069242	PRJEB9562	ERR1223260
3622STDY6094438	Rare	Braenderup	Human	Asymptomatic	PRJEB9121	ERR1046284	PRJEB9562	ERR1223261
2511STDY5908423	Rare	I 4,[5],12:i:-	Human	Blood stream infection	PRJEB1397	ERR702387	PRJEB9562	ERR1223263
10182	Rare	I 4,[5],12:i:-	Human	Diarrhea	PRJEB2973	ERR161957	PRJEB9562	ERR1223264
10413	Rare	Typhimurium	Human	Diarrhea	PRJEB2973	ERR161391	PRJEB9562	ERR1223265
30267	Rare	Typhimurium	Human	Diarrhea	PRJEB2973	ERR161960	PRJEB9562	ERR1223266
617_NTS	Common	I 4,[5],12:i:-	Human	Blood stream infection	PRJEB2973	ERR161454	PRJEB9562	ERR1223267
2511STDY5908468	Common	Typhimurium	Chicken	Asymptomatic	PRJEB1397	ERR715601	PRJEB9562	ERR1223268
3622STDY6094282	Common	Stanley	Human	Diarrhea	PRJEB9121	ERR1069234	PRJEB9562	ERR1223269
3622STDY6094244	Common	Choleraesuis	Human	Blood stream infection	PRJEB9121	ERR1069310	PRJEB9562	ERR1223270
20278	Common	Typhimurium	Human	Diarrhea	PRJEB2973	ERR161438	PRJEB9562	ERR1223271
3622STDY6094321	Common	London	Chicken	Asymptomatic	PRJEB9121	ERR1046173	PRJEB9562	ERR1223272
3622STDY6094275	Common	Panama	Human	Diarrhea	PRJEB9121	ERR1046131	PRJEB9562	ERR1223273
3622STDY6094286	Common	Bovismorbificans	Human	Diarrhea	PRJEB9121	ERR1046141	PRJEB9562	ERR1223274
3622STDY6094263	Common	Panama	Human	Blood stream infection	PRJEB9121	ERR1046121	PRJEB9562	ERR1223275
3622STDY6094249	Common	Schwarzengrund	Human	Blood stream infection	PRJEB9121	ERR1069315	PRJEB9562	ERR1223276
3622STDY6094295	Common	Kedougou	Human	Asymptomatic	PRJEB9121	ERR1046148	PRJEB9562	ERR1223277
198_NTS	Rare	Typhimurium	Human	Blood stream infection	PRJEB2973	ERR161396	PRJEB9562	ERR1223278
3622STDY6094218	Rare	Give	Human	Blood stream infection	PRJEB9121	ERR1069284	PRJEB9562	ERR1223279
3622STDY6094269	Rare	Enteritidis	Human	Blood stream infection	PRJEB9121	ERR1046127	PRJEB9562	ERR1223280
2511STDY5908418	Rare	I 4,[5],12:i:-	Human	Blood stream infection	PRJEB1397	ERR702382	PRJEB9562	ERR1223281
3622STDY6094384	Common	Indiana	Pig	Asymptomatic	PRJEB9121	ERR1046234	PRJEB9562	ERR1223282
10068	Common	Typhimurium	Human	Diarrhea	PRJEB2973	ERR161412	PRJEB9562	ERR1223283
652_NTS	Common	Typhimurium	Human	Blood stream infection	PRJEB2973	ERR161963	PRJEB9562	ERR1223284
2511STDY5908375	Common	I 4,[5],12:i:-	Duck	Asymptomatic	PRJEB1397	ERR702340	PRJEB9562	ERR1223286
1436_NTS	Rare	I 4,[5],12:i:-	Human	Blood stream infection	PRJEB2973	ERR161456	PRJEB9562	ERR1229122
3622STDY6094316	Common	Choleraesuis	Human	Blood stream infection	PRJEB9121	ERR1046169	PRJEB9562	ERR1229123
3622STDY6094199	Common	Panama	Human	Blood stream infection	PRJEB9121	ERR1069266	PRJEB9562	ERR1229124
3622STDY6094380	Common	Senftenberg	Chicken	Asymptomatic	PRJEB9121	ERR1046230	PRJEB9562	ERR1229125
3622STDY6094247	Common	Choleraesuis	Human	Blood stream infection	PRJEB9121	ERR1069313	PRJEB9562	ERR1246953
3622STDY6094333	Common	Derby	Pig	Asymptomatic	PRJEB9121	ERR1046185	PRJEB9562	ERR1246954
3622STDY6094222	Common	Enteritidis	Human	Blood stream infection	PRJEB9121	ERR1069288	PRJEB9562	ERR1246955
2511STDY5462395	Common	Weltevreden	Human	Diarrhea	PRJEB1397	ERR294790	PRJEB9562	ERR1246956
2511STDY5564062	Common	Weltevreden	Duck	Asymptomatic	PRJEB1397	ERR387730	PRJEB9562	ERR1246957
3622STDY6094262	Common	Rissen	Human	Blood stream infection	PRJEB9121	ERR1046120	PRJEB9562	ERR1246958
3622STDY6094320	Common	Indiana	Human	Blood stream infection	PRJEB9121	ERR1069237	PRJEB9562	ERR1246959
10146	Common	I 4,[5],12:i:-	Human	Diarrhea	PRJEB2973	ERR161448	PRJEB9562	ERR1246960
3622STDY6094311	Common	Derby	Human	Asymptomatic	PRJEB9121	ERR1046164	PRJEB9562	ERR1246961
3622STDY6094276	Common	Derby	Human	Diarrhea	PRJEB9121	ERR1046132	PRJEB9562	ERR1246962
1505_NTS	Common	I 4,[5],12:i:-	Human	Blood stream infection	PRJEB2973	ERR161977	PRJEB9562	ERR1246963
1222_NTS	Common	I 4,[5],12:i:-	Human	Blood stream infection	PRJEB2973	ERR161408	PRJEB9562	ERR1246964
3622STDY6094279	Common	Albany	Human	Diarrhea	PRJEB9121	ERR1046135	PRJEB9562	ERR1254193
2511STDY5908462	Common	I 4,[5],12:i:-	Pig	Asymptomatic	PRJEB1397	ERR715595	PRJEB9562	ERR1254194
2511STDY5908324	Common	Typhimurium	Duck	Asymptomatic	PRJEB1397	ERR715548	PRJEB9562	ERR1254195
2511STDY5908457	Common	I 4,[5],12:i:-	Chicken	Asymptomatic	PRJEB1397	ERR715590	PRJEB9562	ERR1254196
3622STDY6094383	Common	Rissen	Pig	Asymptomatic	PRJEB9121	ERR1046233	PRJEB9562	ERR1254197
3622STDY6094387	Common	Indiana	Duck	Asymptomatic	PRJEB9121	ERR1069239	PRJEB9562	ERR1254198
3622STDY6094368	Common	Derby	Pig	Asymptomatic	PRJEB9121	ERR1046218	PRJEB9562	ERR1254199
3622STDY6094344	Common	Derby	Pig	Asymptomatic	PRJEB9121	ERR1046195	PRJEB9562	ERR1254200
3622STDY6094339	Common	Albany	Pig	Asymptomatic	PRJEB9121	ERR1046190	PRJEB9562	ERR1254201
845_NTS	Rare	Typhimurium	Human	Blood stream infection	PRJEB2973	ERR161455	PRJEB9562	ERR1274836
1701_NTS	Rare	I 4,[5],12:i:-	Human	Blood stream infection	PRJEB2973	ERR161435	PRJEB9562	ERR1274837
3622STDY6094207	Rare	Enteritidis	Human	Blood stream infection	PRJEB9121	ERR1069274	PRJEB9562	ERR1274838
3622STDY6094238	Rare	Enteritidis	Human	Blood stream infection	PRJEB9121	ERR1069304	PRJEB9562	ERR1274839

Appendix A. Phenotypic testing comparison

For most isolates, there was concordance between AMR phenotype and genotype (Figure S1). Phenotypic AMR testing determined that the 68 NTS isolates belonged to seventeen phenotypes based on resistance to five antimicrobial classes: aminoglycoside (Am), beta-lactam (Bl), chloramphenicol (Ch), fluoroquinolone (Fl) and trimethoprim (Tr). Of these, twelve consisted of more than one isolate and the genetic elements predicted to be responsible for the phenotypes were compared (Figure S2).

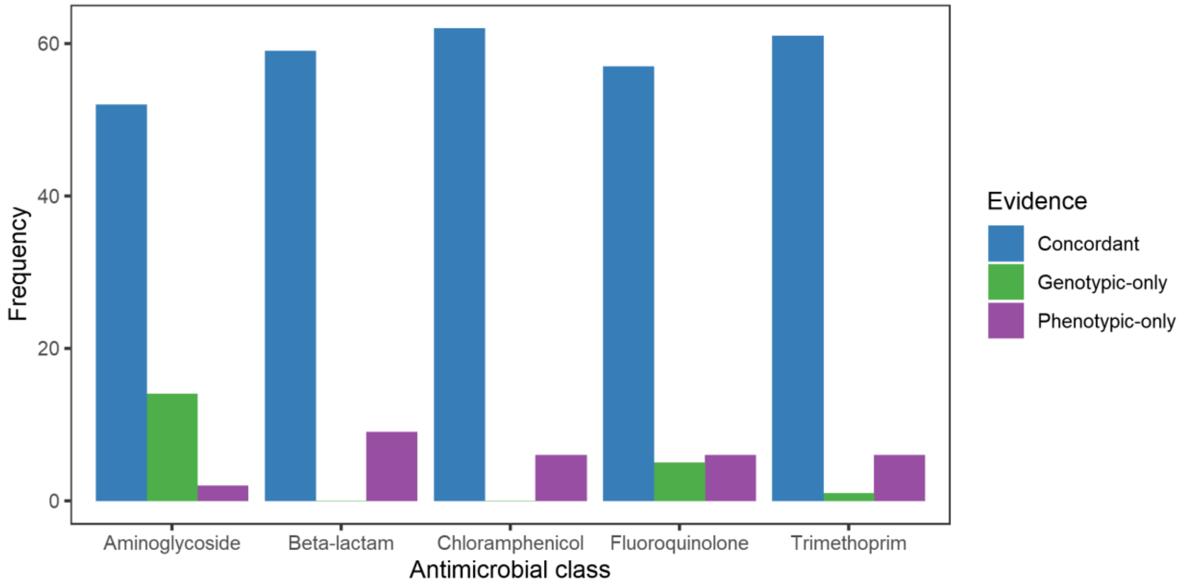

Fig S1. Bar graph of genotypic and phenotypic evidence for 68 NTS isolates regarding five antimicrobial classes.

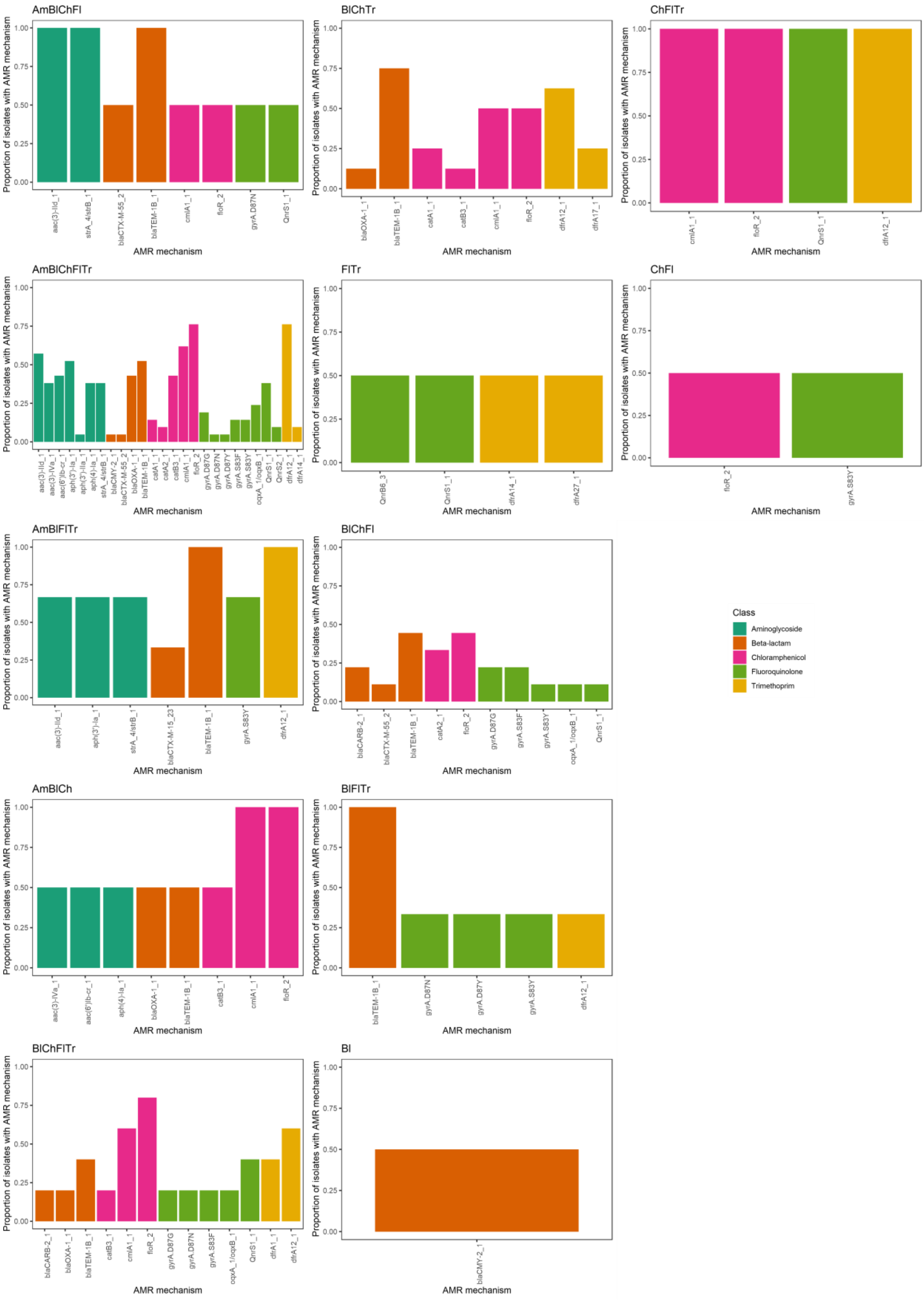

Fig S2. Bar graphs of the proportion of NTS isolates from each AMR phenotype that contain mobile genetic elements predicted to be responsible for their phenotype.

Appendix B. Genes of interest analysis

The genomic locations of AMR, virulence and metal-tolerance genes were compared amongst *Salmonella* from each source, phenotype and infection type (Figure S3). Pan-genome analysis revealed that no AMR genes were included in the core genome and most were found in less than half of the isolates (Figure S4). In contrast, most of the virulence and metal-tolerance genes were found in all isolates. The metal-tolerance genes encoded resistance to 47 metals and antibacterial biocides (Figure S5).

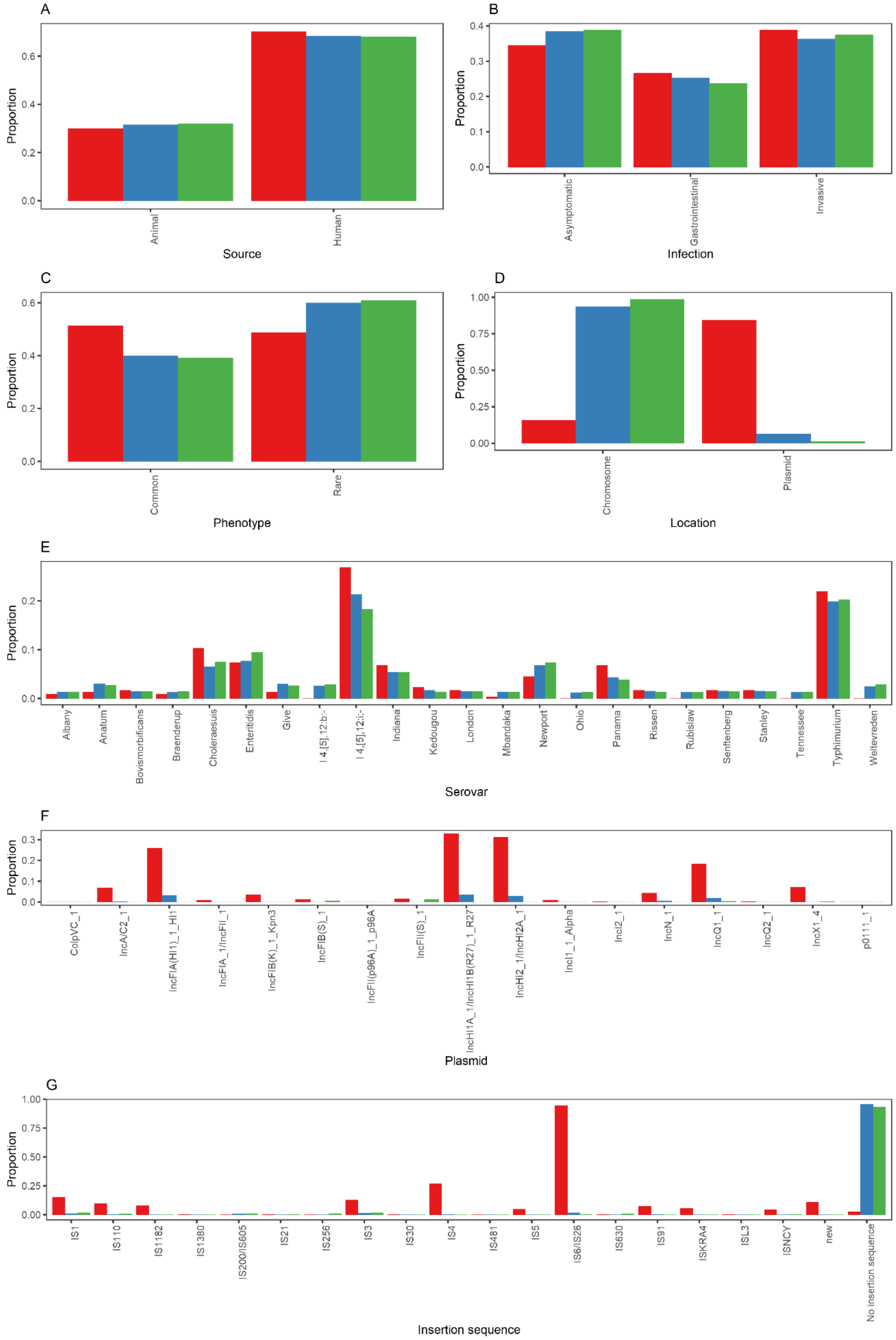

Fig S3. Bar graphs of the proportion of AMR (red), virulence (green) and metal-tolerance (blue) genes found in each source (A), infection type (B), AMR phenotype (C), genome location (D), plasmid type (E) and in proximity to each IS type (F).

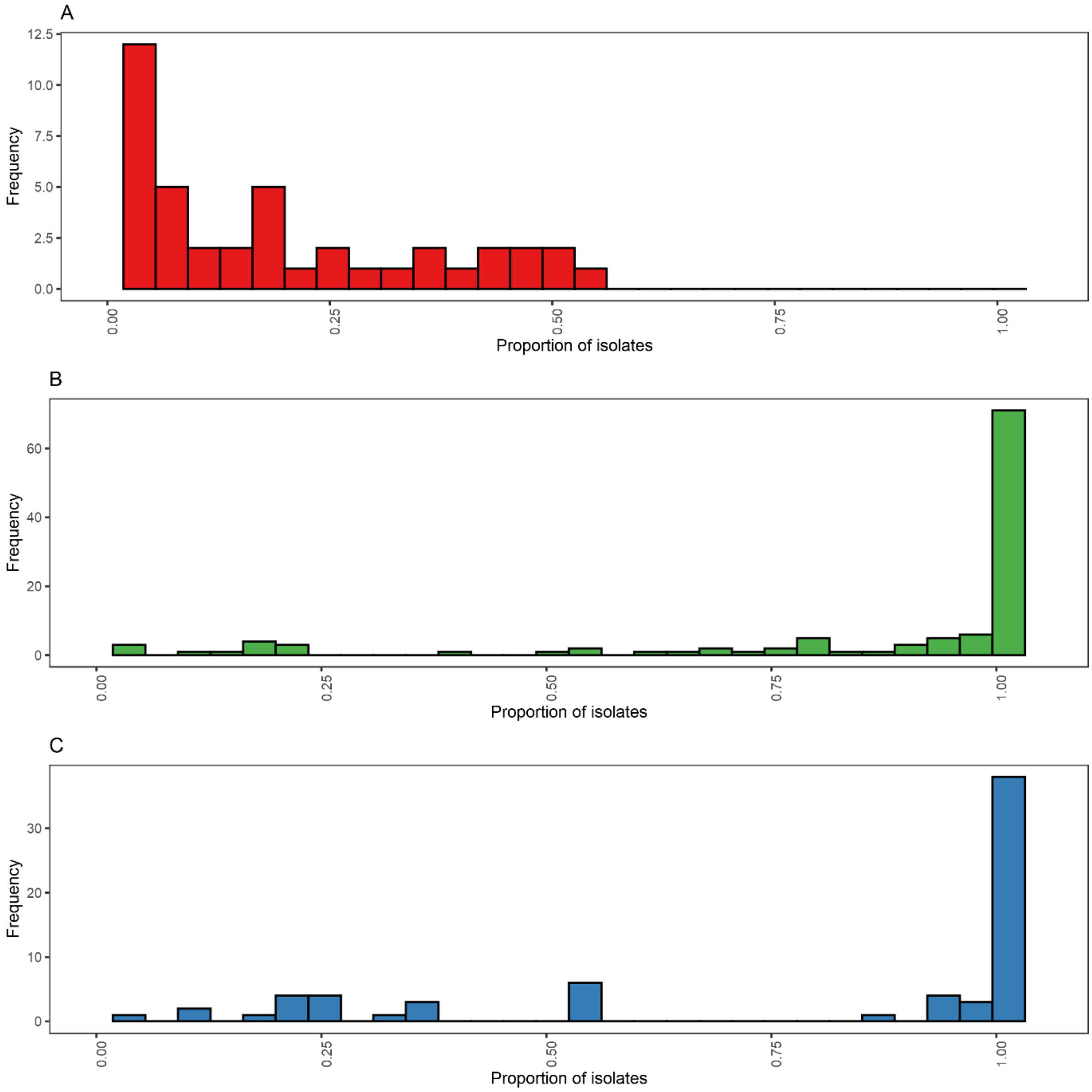

Fig S4. Histograms of the proportion of AMR (A), virulence (B) and metal-tolerance (C) genes found in 68 NTS from Vietnam.

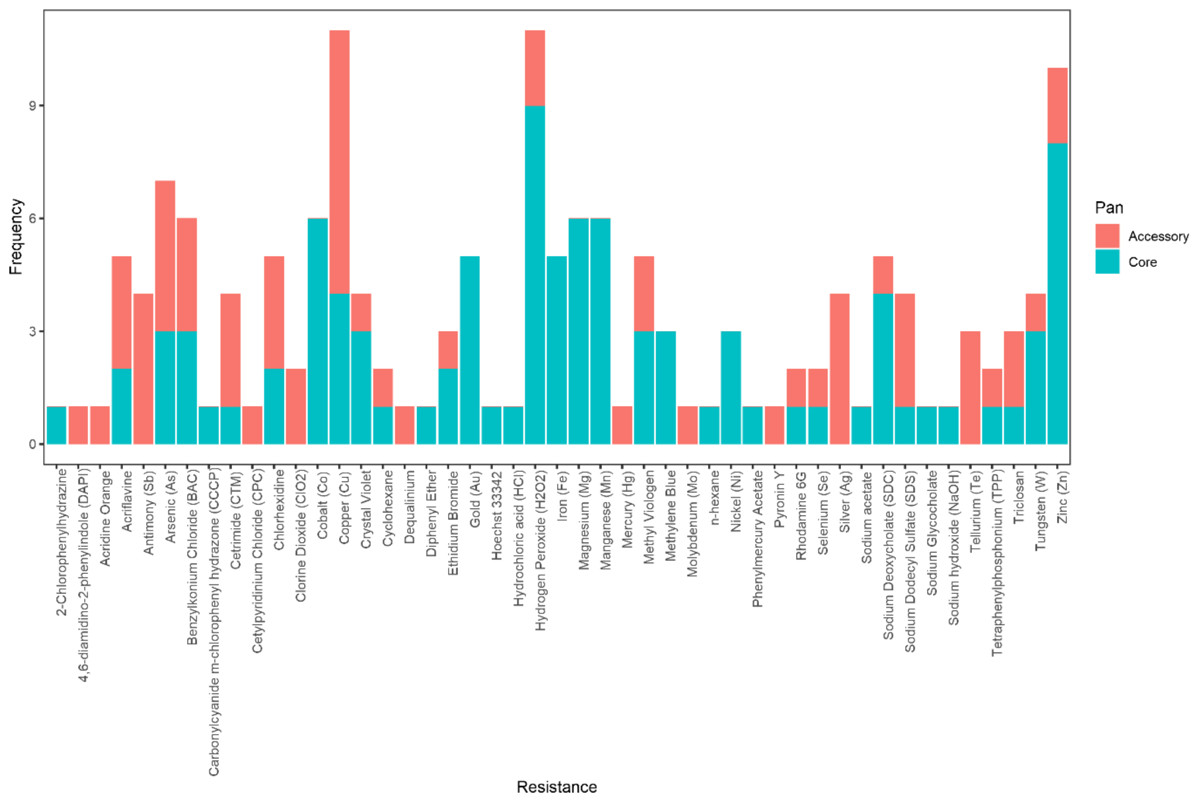

Fig S5. Bar graph of the number of genes encoding resistance to metals and antibacterial biocides and coloured by whether they were core or accessory.

Appendix C. Insertion sequence analysis

Using a cut-off of 10 kb, 98% of AMR genes were close to insertion sequences, compared to 7% of virulence and 5% of metal-tolerance genes. A range of cut-offs were utilized to determine if this result was cut-off dependent (Figure S6). For the 49 AMR gene types identified, six were not found in association with IS6/IS26, but only one to three copies of these gene types were found amongst the 68 NTS isolates investigated (Figure S7). For the remaining AMR genes, 89-100% were associated with IS6/IS26. For the 506 AMR genes located on plasmids, 484 (96%) were associated with IS6/IS26, compared to 83 out of 94 chromosomal AMR genes (88%) (Figure S8). The plasmid contigs containing AMR genes consisted of seventeen different replicon types and five of these did not contain any IS6/IS26-associated AMR, but only one to five copies of these plasmid replicon types were found amongst the 68 NTS isolates investigated (Figure S9). For the remaining plasmid replicon types, 83-100% of AMR genes were associated with IS6/IS26.

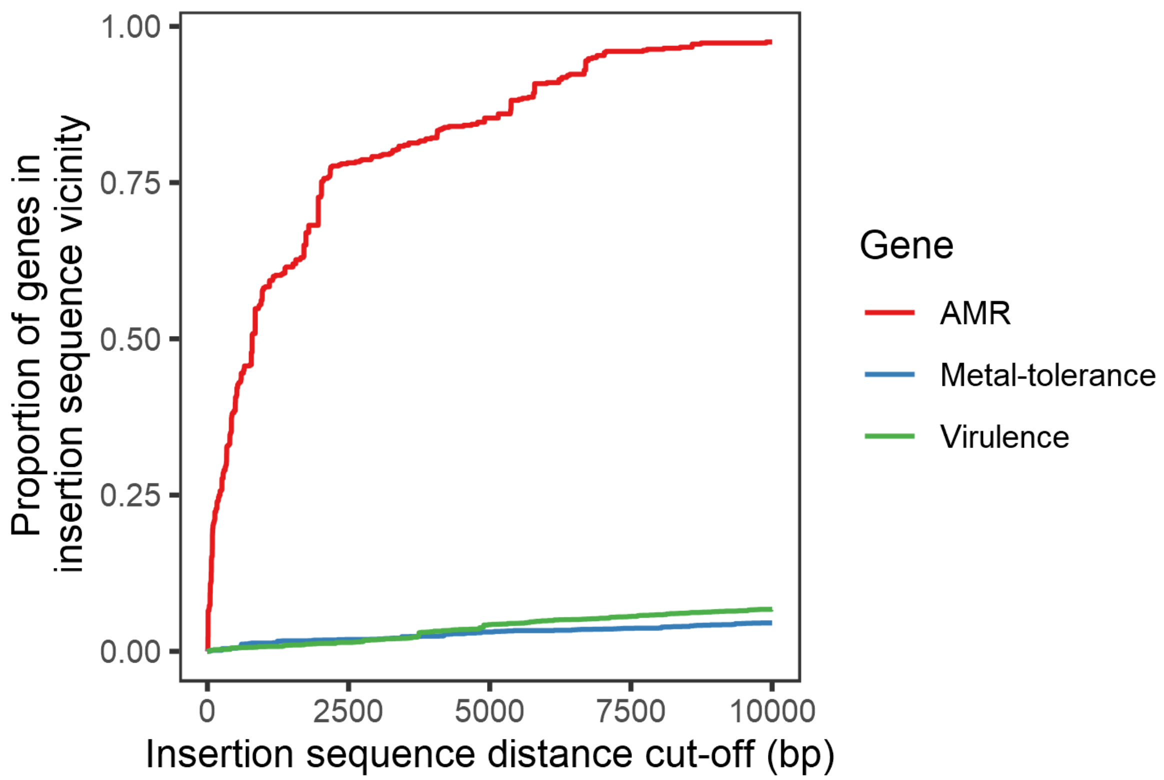

Fig S6. Line graph of the proportion of AMR (red), virulence (green) and metal-tolerance (blue) genes in proximity to IS with different distance cut-offs.

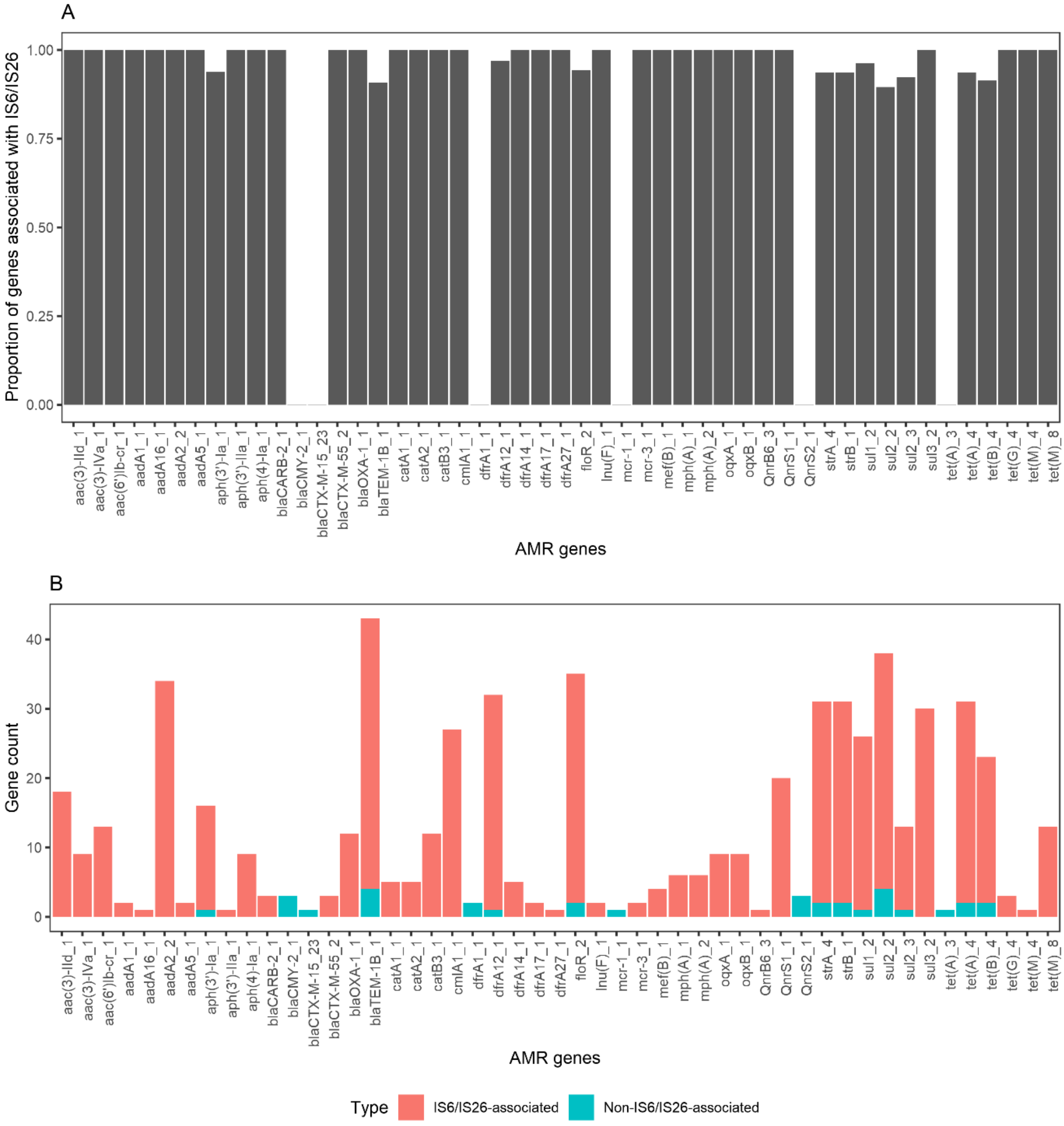

Fig S7. Bar graph of the proportion (A) and number (B) of each AMR gene in association with IS6/IS26 insertion sequences

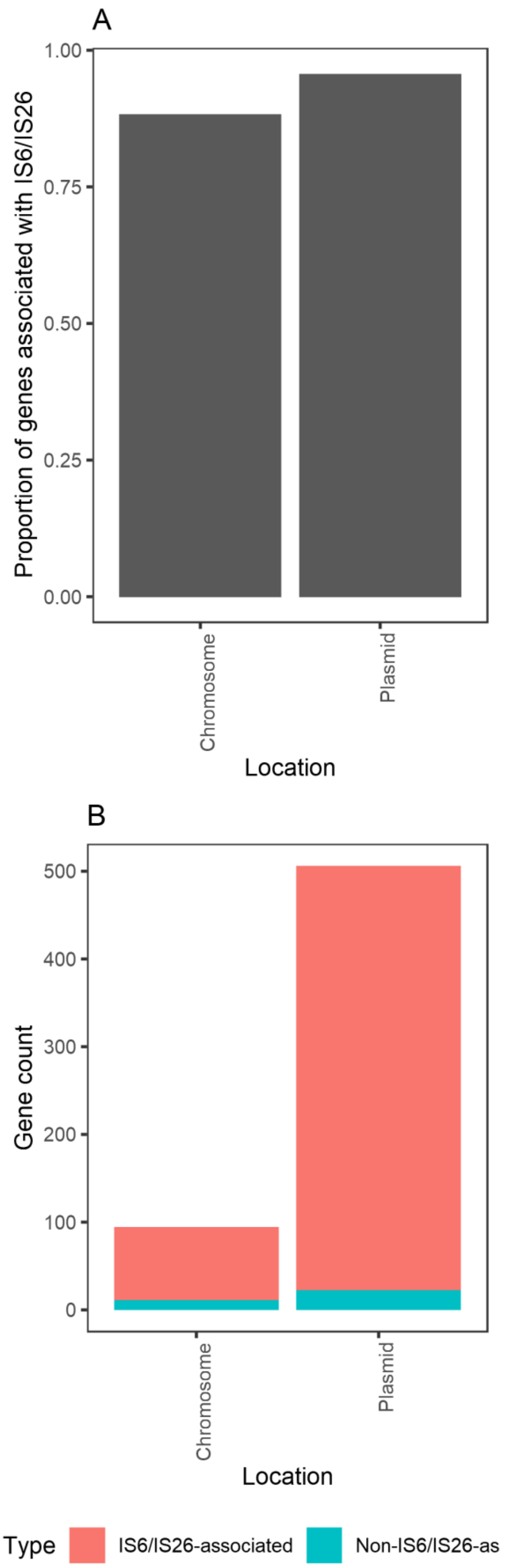

Fig S8. Bar graph of the proportion (A) and number (B) of AMR genes on plasmids and chromosomes associated with IS6/IS26 insertion sequences

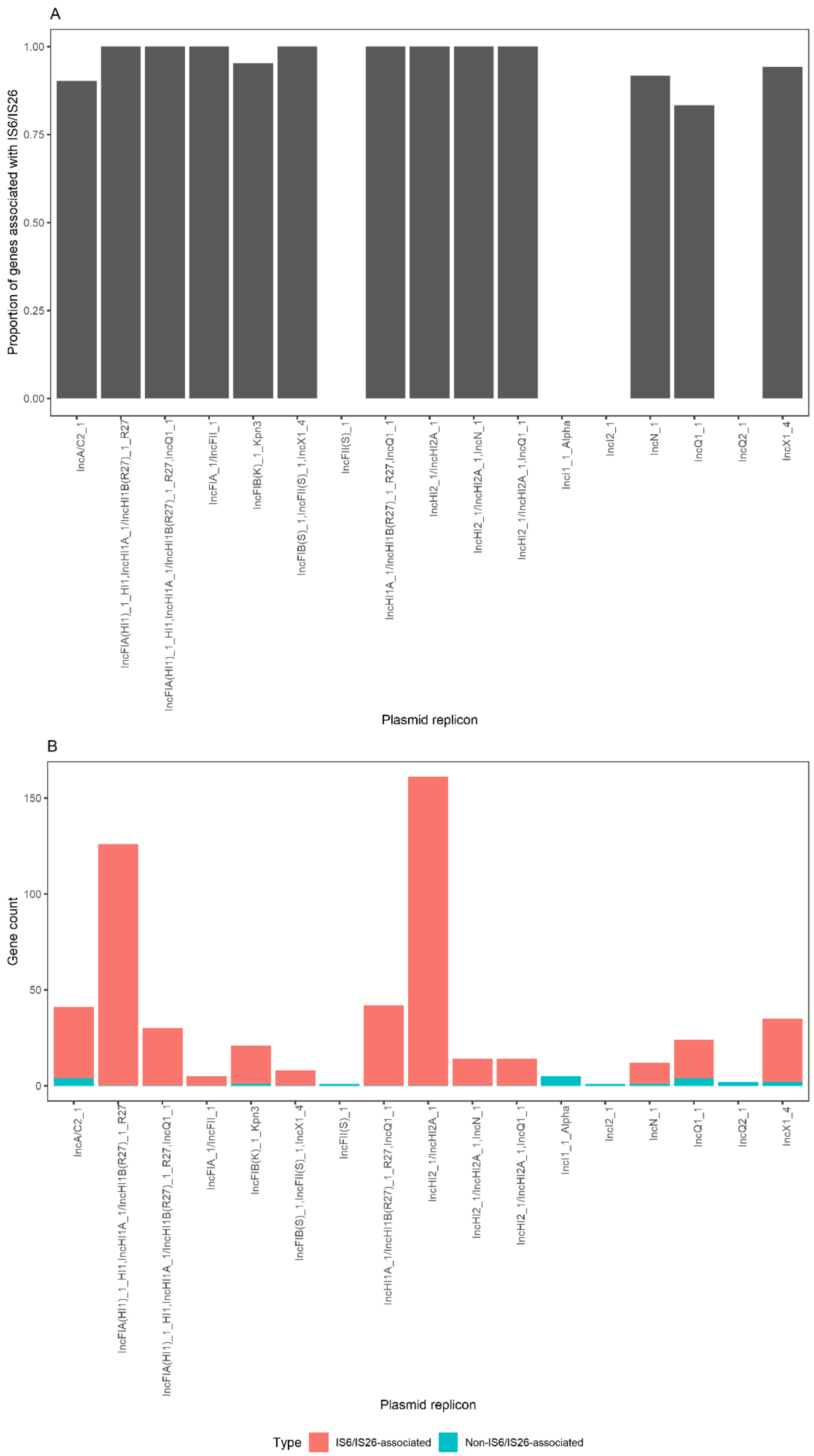

Fig S9. Bar graph of the proportion (A) and number (B) of AMR genes on plasmid contigs with different replicons associated with IS6/IS26 insertion sequences

Appendix D. Phage analysis

Phage analysis identified 162 intact prophage genomes, and of the 1,982 insertion sequences identified, 34 (1.7%) were found on intact prophage genomes (Figure S10). For nineteen of the insertion sequence types, 0-10% were found in intact prophage genomes. For the remaining sequence type (IS481), two out of three of this insertion sequence type were found in intact prophage genomes.

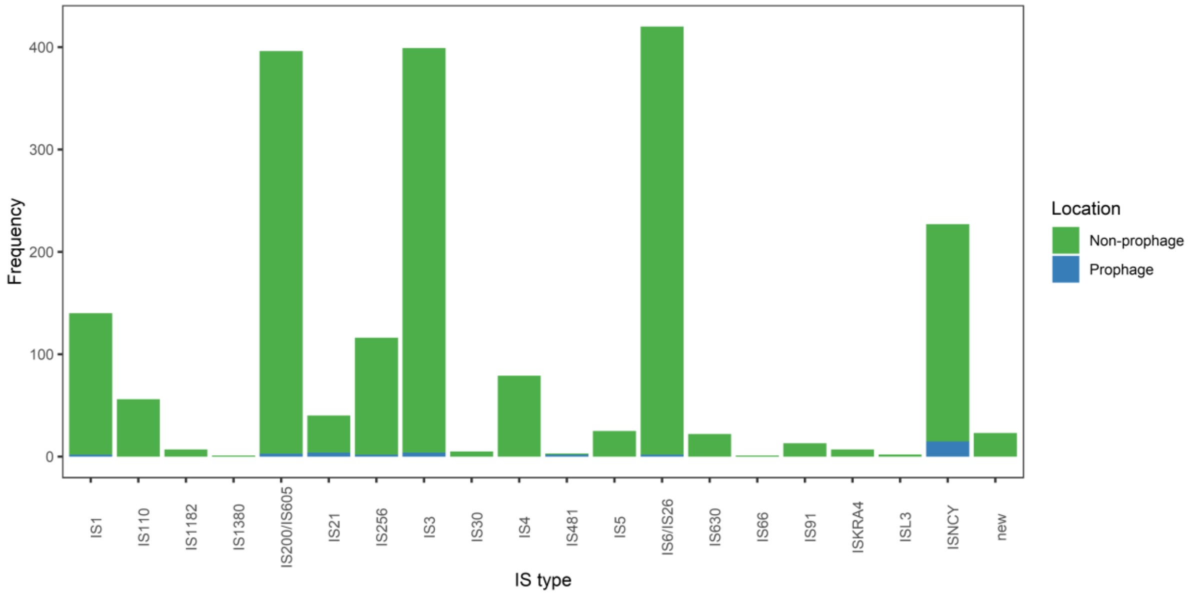

Fig S10. Bar graph of the number of insertion sequence types identified and coloured by the number found in intact prophage genomes.

Appendix E. Plasmid type analysis

For each plasmid type, the contigs containing the plasmid type marker were extracted from the assemblies, aligned, and had their metadata added to look for associations.

The IncHI1A_1/IncHI1B(R27)_1_R27 plasmid type contained the most AMR genes (Figure 2). This plasmid type contained a conserved backbone, and a variable region with a lot of gene variability, including AMR genes and ISs. It also contained a group of metal-tolerance genes that were located in 16/20 of the sequences. The sequences were found in isolates from Bovismorbificans (n=1), Choleraesuis (n=3), Give (n=2), London (n=1), Newport (n=2), Panama (n=3), Stanley (n=1) and Typhimurium (n=7) serovars; animal (n=4) and human (n=16) sources; asymptomatic (n=5), gastrointestinal (n=7) and invasive (n=8) infections; and common (n=12) and rare (n=8) phenotypes. All the sequences were classified as ‘conjugative’.

The IncFIB(K)_1_Kpn3 plasmid type contained a conserved backbone, and a variable region with a lot of gene variability, including AMR genes and ISs (Figure S11). The sequences were found in isolates from Anatum (n=2), Kedougou (n=1) and I,4,[5],12:i:- (n=1) serovars; animal (n=2) and human (n=2) sources; asymptomatic (n=3) and gastrointestinal (n=1) infections; and common (n=2) and rare (n=2) phenotypes. All the sequences were classified as ‘conjugative’.

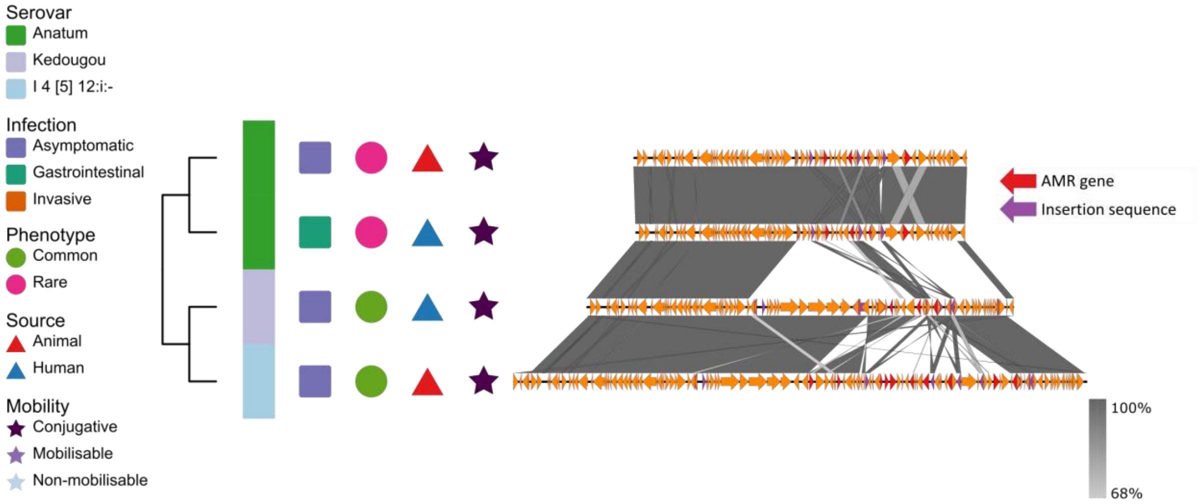

Fig S11. Dendrogram of IncFIB(K)_1_Kpn3 plasmid sequences based on gene presence-absence; symbols representing serovar, type of infection, phenotypic AMR profile commonality, source and plasmid mobility; and alignment of plasmid contigs. Arrows represent genes: red arrows represent AMR genes and purple arrows represent ISs. Bars between contigs represent BLAST alignments.

The IncA_C2_1 plasmid type contained a conserved backbone, and a variable region with a lot of gene variability, including AMR and metal-tolerance genes, and ISs (Figure S12). The sequences were found in isolates from Choleraesuis (n=1), Rissen (n=1) and I,4,[5],12:i:- (n=2) serovars; animal (n=2) and human (n=2) sources; asymptomatic (n=2) and invasive (n=2) infections; and common (n=2) and rare (n=2) phenotypes. All the sequences were classified as ‘conjugative’.

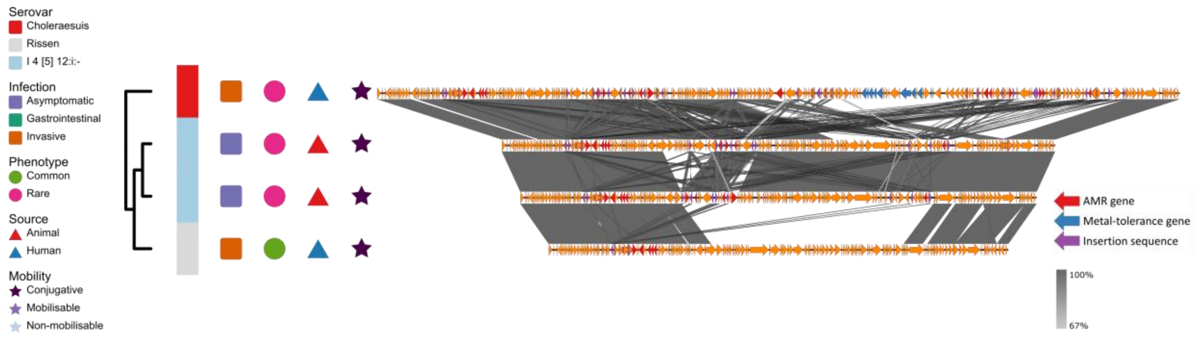

Fig S12. Dendrogram of IncA_C2_1 plasmid sequences based on gene presence-absence; symbols representing serovar, type of infection, phenotypic AMR profile commonality, source and plasmid mobility; and alignment of plasmid contigs. Arrows represent genes: red arrows represent AMR genes, blue represent metal-tolerance genes and purple arrows represent ISs. Bars between contigs represent BLAST alignments.

The IncHI2_1/IncHI2A_1 plasmid type contained many AMR genes alongside insertion sequences throughout the plasmid (Figure S13). Of the sixteen sequences containing the IncHI2_1/IncHI2A_1 plasmid type, one was inserted into the chromosome, whilst the rest were not. The sequences were found in isolates from Indiana (n=2), Kedougou (n=1), Mbandaka (n=1), Typhimurium (n=4) and I,4,[5],12:i:- (n=8) serovars; animal (n=5) and human (n=11) sources; asymptomatic (n=6), gastrointestinal (n=5) and invasive (n=5) infections; and common (n=8) and rare (n=8) phenotypes. The sequences were classified as ‘conjugative’ (n=10) or ‘non-mobilizable’ (n=6).

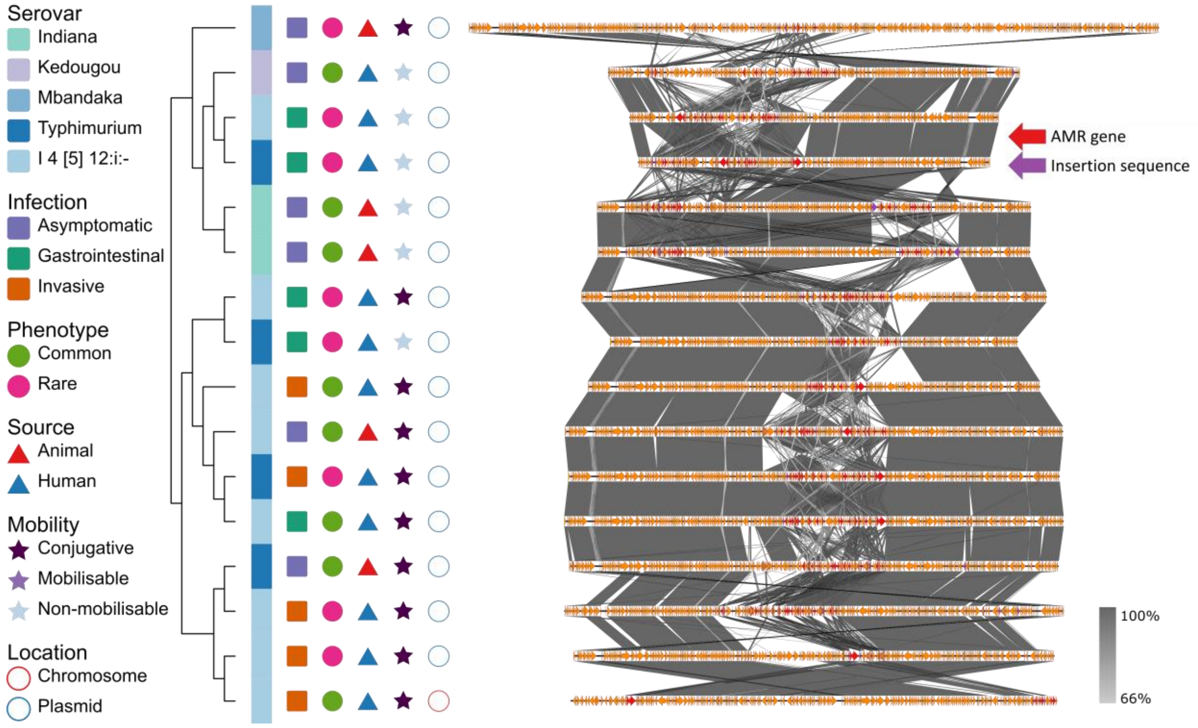

Fig S13. Dendrogram of IncHI2_1/IncHI2A_1 plasmid sequences based on gene presence-absence; symbols representing serovar, type of infection, phenotypic AMR profile commonality, source, location of plasmid and plasmid mobility; and alignment of plasmid contigs. Arrows represent genes: red arrows represent AMR genes and purple arrows represent ISs. Bars between contigs represent BLAST alignments.

The IncQ1_1 plasmid type contained many AMR genes alongside insertion sequences, along with virulence and metal-tolerance genes throughout the plasmid (Figure S14). Of the fourteen sequences containing the IncQ1_1 plasmid type, four were inserted into the chromosome, whilst the rest were not. The sequences were found in isolates from Choleraesuis (n=4), Typhimurium (n=5) and I,4,[5],12:i:- (n=5) serovars; animal (n=3) and human (n=11) sources; asymptomatic (n=3), gastrointestinal (n=3) and invasive (n=8) infections; and common (n=5) and rare (n=9) phenotypes. The sequences were classified as ‘conjugative’ (n=7), ‘mobilizable’ (n=5) or ‘non-mobilizable’ (n=2).

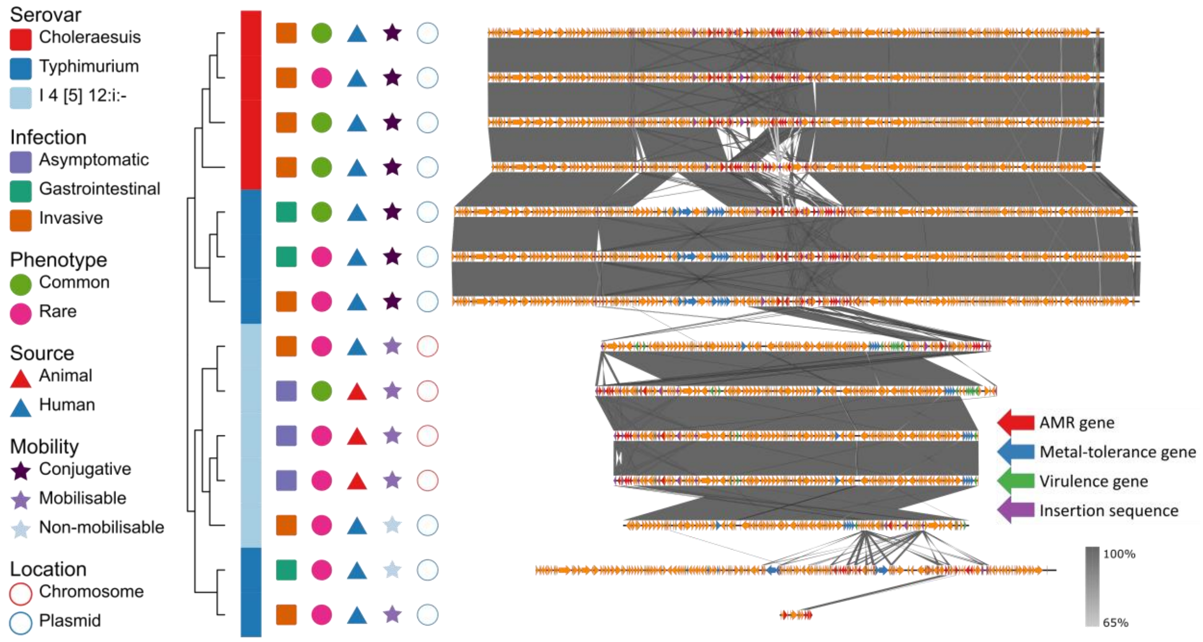

Fig S14. Dendrogram of IncQ1_1 plasmid sequences based on gene presence-absence; symbols representing serovar, type of infection, phenotypic AMR profile commonality, source, location of plasmid and plasmid mobility; and alignment of plasmid contigs. Arrows represent genes: red arrows represent AMR genes, green arrows represent virulence genes, blue arrows represent metal-tolerance genes and purple arrows represent ISs. Bars between contigs represent BLAST alignments.

The IncX1 plasmid type contained many AMR genes alongside insertion sequences, along with virulence genes throughout the plasmid (Figure S15). Of the seven sequences containing the IncX1 plasmid type, three were inserted into the chromosome, whilst the rest were not. The sequences were found in isolates from Enteritidis (n=6) and Typhimurium (n=1) serovars; animal (n=2) and human (n=5) sources; asymptomatic (n=2) and invasive (n=5) infections; and common (n=2) and rare (n=5) phenotypes. The sequences were classified as ‘mobilizable’ (n=1) or ‘non-mobilizable’ (n=6).

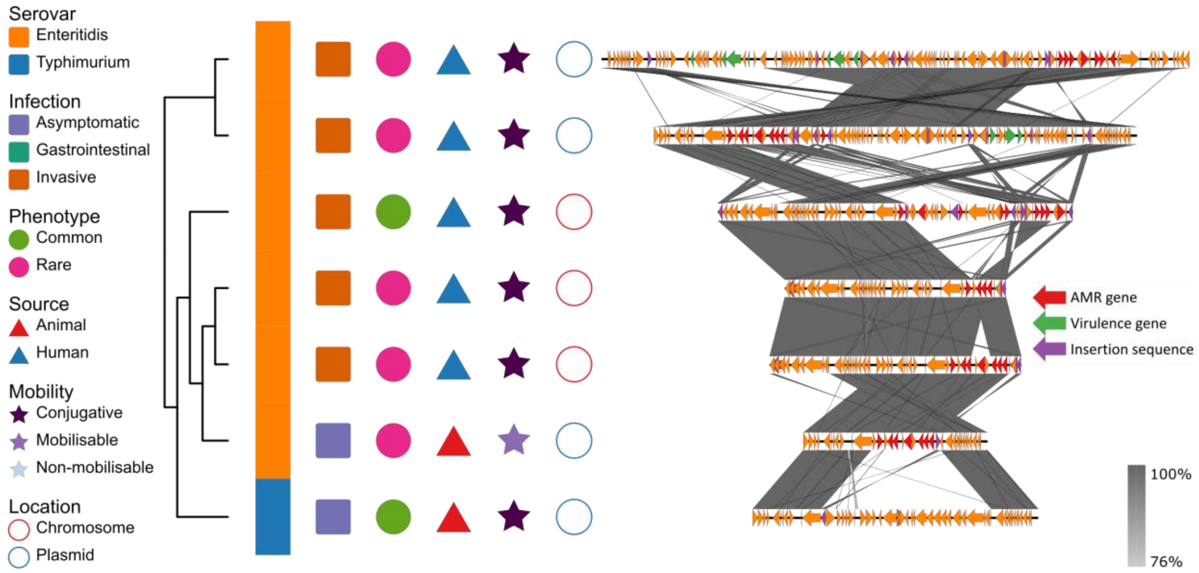

Fig S15. Dendrogram of IncX1 plasmid sequences based on gene presence-absence; symbols representing serovar, type of infection, phenotypic AMR profile commonality, source, location of plasmid and plasmid mobility; and alignment of plasmid contigs. Arrows represent genes: red arrows represent AMR genes, green arrows represent virulence genes and purple arrows represent ISs. Bars between contigs represent BLAST alignments.

The IncFII(S)_1 plasmid type contained virulence genes in 13 out of 15 sequences, and AMR genes in two sequences (Figure 4). The sequences were found in isolates from Choleraesuis (n=5), Enteritidis (n=5), Typhimurium (n=3) and Weltevreden (n=2) serovars; animal (n=3) and human (n=12) sources; asymptomatic (n=3), gastrointestinal (n=1) and invasive (n=11) infections; and common (n=6) and rare (n=9) phenotypes. The sequences were classified as 'conjugative' (n=8), 'mobilizable' (n=3) or 'non-mobilizable' (n=4).

The IncI1_1 plasmid type contained AMR genes in five out of six sequences, and virulence genes in one sequence (Figure S16). The sequences were found in isolates from Albany (n=1), Newport (n=1), Typhimurium (n=2) and I,4,[5],12:i:- (n=2) serovars; animal (n=1) and human (n=5) sources; asymptomatic (n=1), gastrointestinal (n=4) and invasive (n=1) infections; and common (n=1) and rare (n=5) phenotypes. The sequences were classified as 'conjugative' (n=5) or 'mobilizable' (n=1).

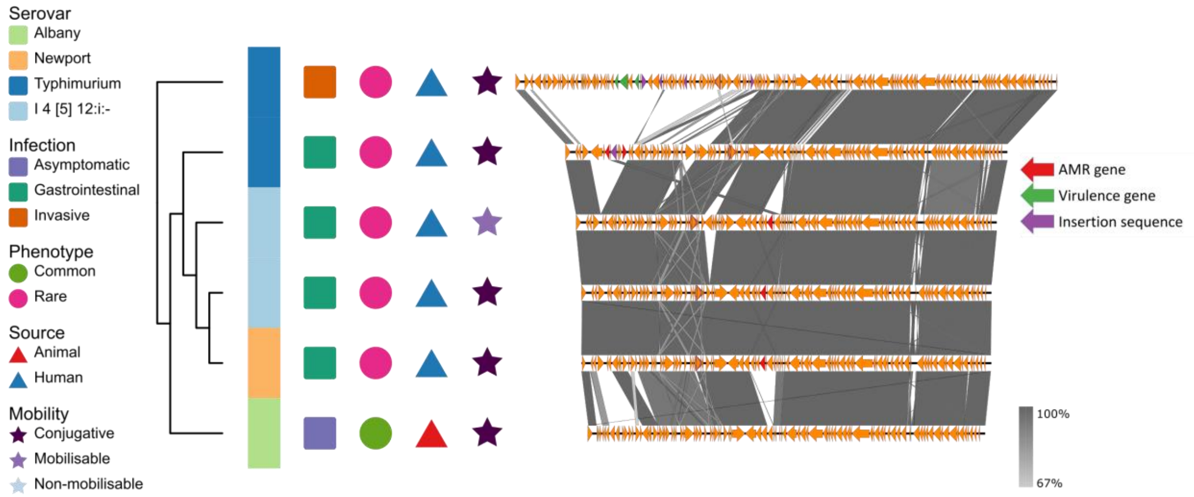

Fig S16. Dendrogram of IncI1_1 plasmid sequences based on gene presence-absence; symbols representing serovar, type of infection, phenotypic AMR profile commonality, source and plasmid mobility; and alignment of plasmid contigs. Arrows represent genes: red arrows represent AMR genes, green arrows represent virulence genes and purple arrows represent ISs. Bars between contigs represent BLAST alignments.

The IncN_1 plasmid type contained AMR genes in three out of four sequences, and metal-tolerance genes in two sequences (Figure S17). The sequences varied in gene content and size, with no conserved regions. The sequences were found in isolates from Braenderup (n=1), Newport (n=1) and I,4,[5],12:i:- (n=2) serovars; animal (n=1) and human (n=3) sources; asymptomatic (n=2) and invasive (n=2) infections; and the rare phenotype (n=4). The sequences were all classified as ‘conjugative’.

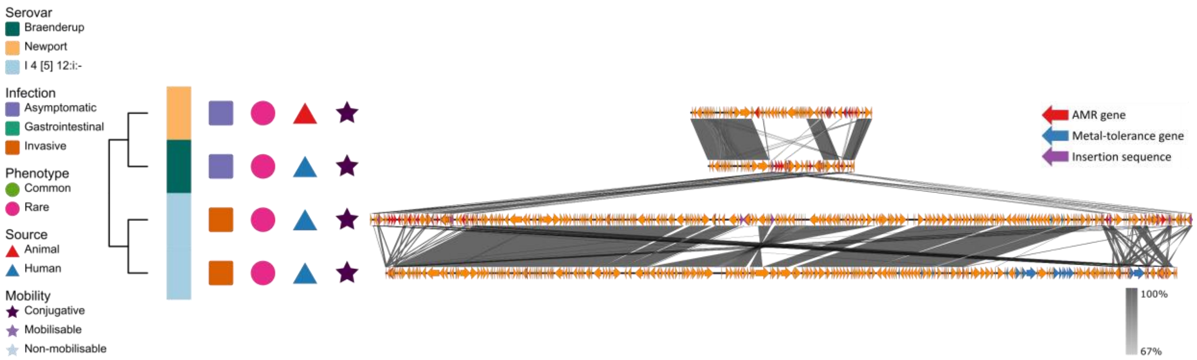

Fig S17. Dendrogram of IncN_1 plasmid sequences based on gene presence-absence; symbols representing serovar, type of infection, phenotypic AMR profile commonality, source and plasmid mobility; and alignment of plasmid contigs. Arrows represent genes: red arrows represent AMR genes, blue arrows represent metal-tolerance genes and purple arrows represent ISs. Bars between contigs represent BLAST alignments.

The IncQ2 plasmid type consisted of two sequences, one of which contained AMR genes (Figure S18). The sequences were all 'mobilizable' and from I 4,[5],12:i:- isolates with common phenotypes, but they found in animal (n=1) and human (n=1) sources; and asymptomatic (n=1) and gastrointestinal (n=1) infections.

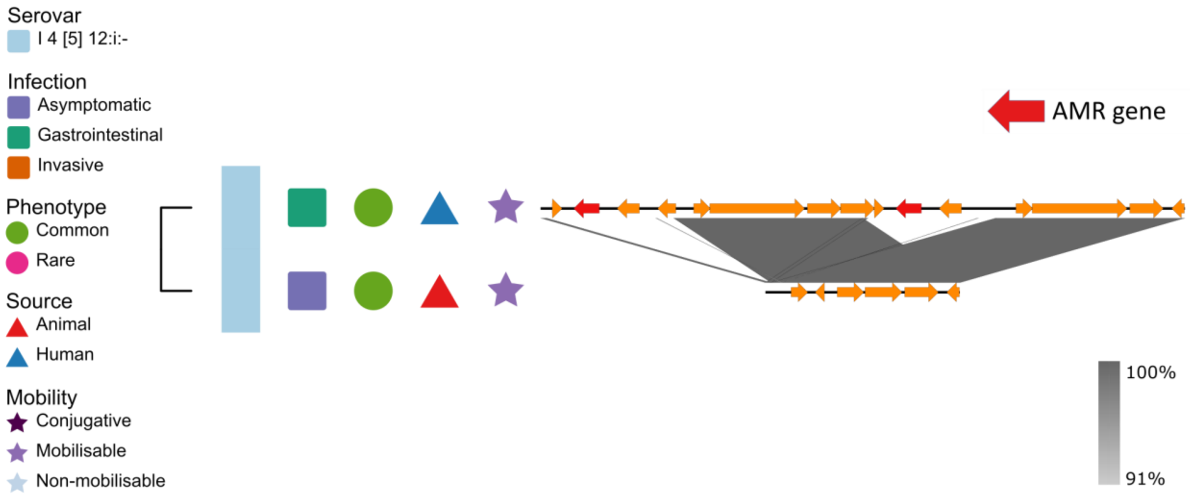

Fig S18. Dendrogram of IncQ2 plasmid sequences based on gene presence-absence; symbols representing serovar, type of infection, phenotypic AMR profile commonality, source and plasmid mobility; and alignment of plasmid contigs. Arrows represent genes: red arrows represent AMR genes. Bars between contigs represent BLAST alignments.

The p0111_1 plasmid type consisted of two sequences, none of which contained AMR, virulence, or metal-tolerance genes (Figure S19). The sequences were all ‘non-mobilizable’ and from Indiana isolates, and were found in animal (n=1) and human (n=1) sources; asymptomatic (n=1) and invasive (n=1) infections; and common (n=1) and rare (n=1) phenotype.

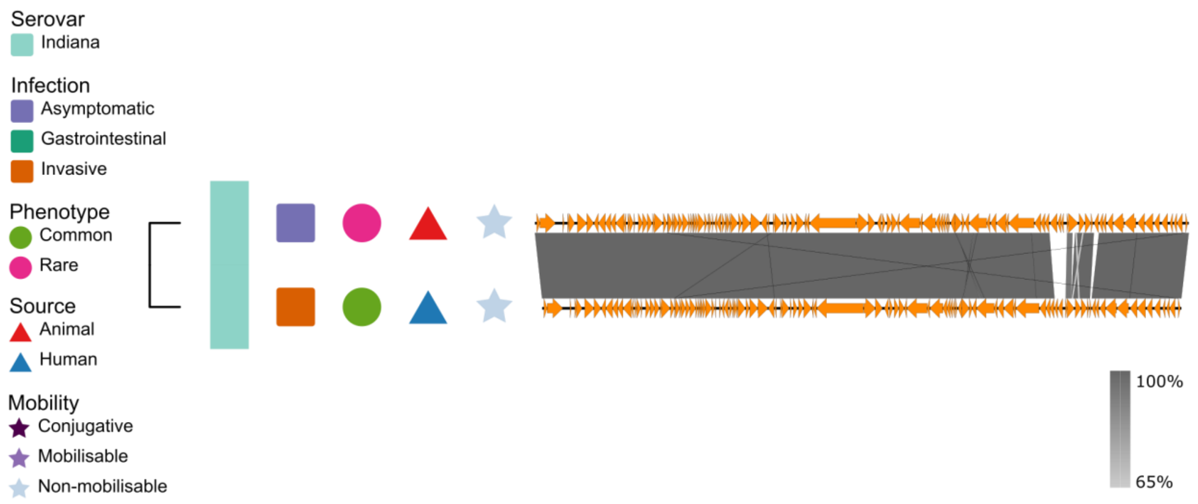

Fig S19. Dendrogram of p0111_1 plasmid sequences based on gene presence-absence; symbols representing serovar, type of infection, phenotypic AMR profile commonality, source and plasmid mobility; and alignment of plasmid contigs. Arrows represent genes. Bars between contigs represent BLAST alignments.

The IncFIA_1/IncFII_1 plasmid type consisted of one sequence that contained AMR genes in close proximity to insertion sequences. The sequence was ‘conjugative’ and from a Typhimurium isolate with a rare phenotype from a human with an invasive infection.

The IncFII(p96A)_1_p96A plasmid type consisted of one sequence that did not contain any AMR, virulence or metal-tolerance genes. The sequence was ‘non-mobilizable’ and from an Ohio isolate with a rare phenotype from an asymptomatic human.

The IncI2_1 plasmid type consisted of one sequence that contained one AMR gene, but not virulence or metal-tolerance genes. The sequence was ‘conjugative’ and from a Newport isolate with a rare phenotype from a human with a gastrointestinal infection.

The ColpVC 1_p96A plasmid type consisted of one sequence that did not contain any AMR, virulence, or metal-tolerance genes. The sequence was ‘non-mobilizable’ and from an Albany isolate with a common phenotype from an asymptomatic animal.

Linear regression models were used to model the total number of AMR, virulence and metal-tolerance genes with the isolate metadata, plasmid type and the number of each IS type on the plasmid sequences. Partial-F tests were used to determine if the categorical variables significantly improved the fit of the model. AMR genes fitted the model better than virulence or metal-tolerance genes as a larger proportion of plasmid sequences contained AMR genes than virulence or metal-tolerance genes. The frequency of IS6/IS26 insertion sequences along plasmid sequences was positively associated with the number of AMR genes on these sequences (Figure S20). However, some insertion sequence and replicon types consisted of one observation, resulting in no statistics for these variables (“NA” below).

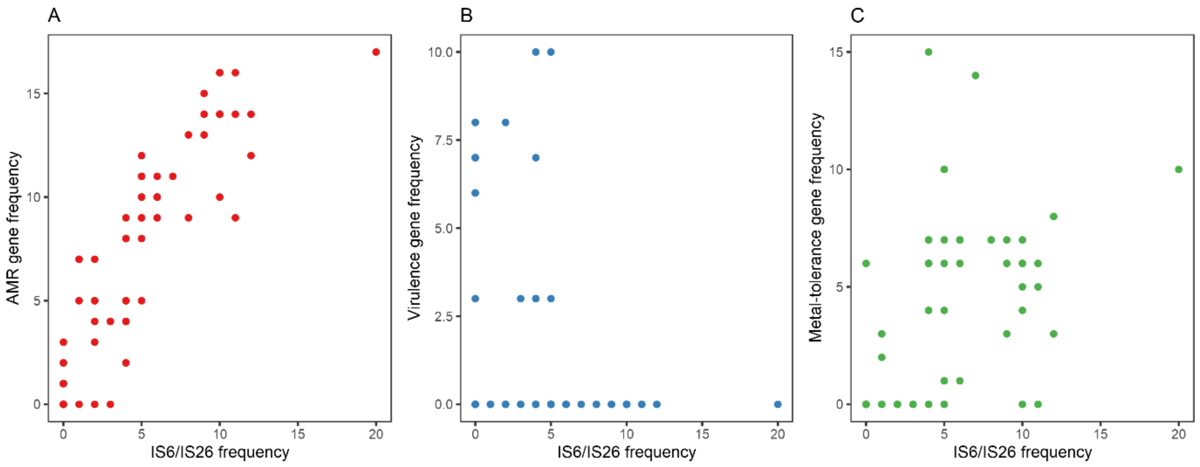

Fig S20. Scatterplot of the number of AMR (A), metal-tolerance (B) and virulence (C) genes versus the number of IS6/IS26 insertion sequences on plasmid sequences.

AMR gene plasmid linear regression model

Linear regression formula:

AMR ~ Factor(Mobility) + Factor(Serovar) + Factor(Source) + Factor(Infection) + Factor(Phenotype) + IncQ1_1 + IncA_C2_1 + p0111_1 + IncFIB(K)_1_Kpn3 + IncFIA(HI1)_1_HI1 + IncHI1A_1/IncHI1B(R27)_1_R27 + IncFII(S)_1 + IncHI2_1/IncHI2A_1 + IncFIB(S)_1 + IncI1_1_Alpha + IncI2_1 + IncFII(p96A)_1_p96A + IncX1_4 + IncN_1 + IncFIA_1_IncFII_1 + IncQ2_1 + ColpVC_1 + ISNCY + IS200_IS605 + IS6/IS26 + IS4 + IS1 + IS256 + IS3 + IS91 + IS1182 + IS630 + IS30 + IS21 + IS5 + IS110 + ISL3 + new + IS1380 + ISKRA4 + IS66

Residuals:

Minimum	First quartile	Median	Third quartile	Maximum
-2.759	-0.3704	0.0000	0.3458	2.922

Coefficient	Estimate	Standard error	t-value	p-value
**Intercept**	2.718	2.844	0.956	0.3470
**Mobility-Mobilizable**	1.145	1.242	0.922	0.3641
**Mobility-Non-mobilizable**	-1.085	1.396	-0.777	0.4434
**Serovar-Anatum**	-2.328	4.706	-0.495	0.6246
**Serovar-Bovismorbificans**	-0.1243	3.373	$-3.7x10^{-2}$	0.9709
**Serovar-Braenderup**	5.280	6.036	0.875	0.3889
**Serovar-Choleraesuis**	0.9319	2.821	0.33	0.7435
**Serovar-Enteritidis**	2.484	3.107	0.8	0.4304
**Serovar-Give**	-3.861	3.386	-1.14	0.2635
**Serovar-I 4,[5],12:i:-**	-1.302	2.594	-0.502	0.6197
**Serovar-Indiana**	-6.092	5.098	-1.195	0.2418
**Serovar-Kedougou**	-5.910	6.093	-0.97	0.3401
**Serovar-London**	2.279	3.206	0.711	0.4829
**Serovar-Mbandaka**	-12.38	6.136	-2.018	$5.291x10^{-2}$
**Serovar-Newport**	0.1148	2.766	$4.1x10^{-2}$	0.9672
**Serovar-Ohio**	-1.104	4.256	-0.259	0.7972
**Serovar-Panama**	0.4964	3.301	0.15	0.8815
**Serovar-Rissen**	1.342	3.989	0.337	0.7389
**Serovar-Stanley**	-0.1243	3.373	$-3.7x10^{-2}$	0.9709
**Serovar-Typhimurium**	0.1270	2.639	$4.8x10^{-2}$	0.9620
**Serovar-Weltevreden**	8.736	10.24	0.853	0.4004
**Source-Human**	-1.130	2.184	-0.518	0.6086
**Infection-Gastrointestinal**	1.840	2.277	0.808	0.4257
**Infection-Invasive**	0.2853	2.350	0.121	0.9042
**Phenotype-Rare**	0.6011	0.6200	0.97	0.3402
**IncQ1_1**	-2.017	1.932	-1.044	0.3053

**IncA_C2_1**	-3.417	7.124	-0.48	0.6351
**p0111_1**	4.581	4.599	0.996	0.3274
**IncFIB(K)_1_Kpn3**	$7.023x10^{-2}$	3.203	$2.2x10^{-2}$	0.9827
**IncFIA(HI1)_1_HI1**	-1.528	1.807	-0.845	0.4048
**IncHI1A_1/IncHI1B(R27)_1_R27**	7.099	4.593	1.545	0.1331
**IncFII(S)_1**	-2.730	2.084	-1.31	0.2004
**IncHI2_1/IncHI2A_1**	3.422	2.580	1.326	0.1951
**IncFIB(S)_1**	0.8036	1.182	0.68	0.5020
**IncI1_1_Alpha**	-2.718	2.304	-1.18	0.2477
**IncI2_1**	-3.144	2.881	-1.091	0.2842
**IncFII(p96A)_1_p96A**	NA	NA	NA	NA
**IncX1_4**	-2.248	2.239	-1.004	0.3238
**IncN_1**	$3.653x10^{-2}$	2.365	$1.5x10^{-2}$	0.9878
**IncFIA_1/IncFII_1**	4.929	3.430	1.437	0.1615
**IncQ2_1**	-1.917	2.362	-0.811	0.4237
**ColpVC_1**	-1.633	3.574	-0.457	0.6511
**ISNCY**	-0.3365	1.567	-0.215	0.8315
**IS200_IS605**	-6.344	4.451	-1.425	0.1647
**IS6/IS26**	0.7699	0.2216	3.474	$1.63x10^{-3}$
**IS4**	1.033	0.8758	1.18	0.2477
**IS1**	0.2737	0.9754	0.281	0.781
**IS256**	NA	NA	NA	NA
**IS3**	0.1619	0.8031	0.202	0.8416
**IS91**	3.662	6.724	0.545	0.5902
**IS1182**	-3.574	2.451	-1.458	0.1557
**IS630**	-0.4363	1.472	-0.296	0.7691
**IS30**	NA	NA	NA	NA
**IS21**	-1.693	1.079	-1.569	0.1275
**IS5**	1.852	2.189	0.846	0.4044
**IS110**	2.273	2.067	1.1	0.2805
**ISL3**	-6.230	7.741	-0.805	0.4275
**New**	1.285	1.137	1.131	0.2674
**IS1380**	0.8987	2.132	0.421	0.6765
**ISKRA4**	-3.854	3.431	-1.123	0.2705
**IS66**	-6.142	7.507	-0.818	0.4200

**Statistic**	**Value**
**Residual standard error**	1.667 on 29 degrees of freedom
**Multiple R-squared**	0.9665
**Adjusted R-squared**	0.9008
**F-statistic**	14.7 on 57 and 29 degrees of freedom
**p-value**	$1.234x10^{-11}$

Partial-F test mobility

Model	Residual degrees of freedom	Residual sum of squares	Degrees of freedom	Sum of squares	F-value	p-value
**AMR plasmid**	29	80.54				
**AMR plasmid minus mobility**	31	88.64	-2	-8.101	1.456	0.2492

Partial-F test serovar

Model	Residual degrees of freedom	Residual sum of squares	Degrees of freedom	Sum of squares	F-value	p-value
**AMR plasmid**	29	80.54				
**AMR plasmid minus serovar**	45	153.1	-16	-72.55	1.633	0.1222

Partial-F test infection

Model	Residual degrees of freedom	Residual sum of squares	Degrees of freedom	Sum of squares	F-value	p-value
**AMR plasmid**	29	80.54				
**AMR plasmid minus infection**	47	156.6	-18	-76.04	1.521	0.153

Partial-F test phenotype

Model	Residual degrees of freedom	Residual sum of squares	Degrees of freedom	Sum of squares	F-value	p-value
**AMR plasmid**	29	80.54				
**AMR plasmid minus phenotype**	32	91.19	-3	-10.65	1.278	0.3005

Partial-F test source

Model	Residual degrees of freedom	Residual sum of squares	Degrees of freedom	Sum of squares	F-value	p-value
**AMR plasmid**	29	80.54				
**AMR plasmid minus source**	32	89.20	-3	-8.660	1.040	0.3898

Partial-F test plasmid type

Model	Residual degrees of freedom	Residual sum of squares	Degrees of freedom	Sum of squares	F-value	p-value
**AMR plasmid**	29	80.54				
**AMR plasmid minus plasmid type**	44	129.0	-15	-48.50	1.164	0.3503

Virulence gene plasmid linear regression model

Linear regression formula:

Virulence ~ Factor(Mobility) + Factor(Serovar) + Factor(Source) + Factor(Infection) + Factor(Phenotype) + IncQ1_1 + IncA_C2_1 + p0111_1 + IncFIB(K)_1_Kpn3 + IncFIA(HI1)_1_HI1 + IncHI1A_1/IncHI1B(R27)_1_R27 + IncFII(S)_1 + IncHI2_1/IncHI2A_1 + IncFIB(S)_1 + IncI1_1_Alpha + IncI2_1 + IncFII(p96A)_1_p96A + IncX1_4 + IncN_1 + IncFIA_1_IncFII_1 + IncQ2_1 + ColpVC_1 + ISNCY + IS200_IS605 + IS6/IS26 + IS4 + IS1 + IS256 + IS3 + IS91 + IS1182 + IS630 + IS30 + IS21 + IS5 + IS110 + ISL3 + new + IS1380 + ISKRA4 + IS66

Residuals:

Minimum	First quartile	Median	Third quartile	Maximum
-2.418	-0.2105	0.0000	0.1191	4.440

Coefficient	Estimate	Standard error	t-value	p-value
**Intercept**	1.258	2.393	0.526	0.6031
**Mobility-Mobilizable**	0.1887	1.045	0.18	0.858
**Mobility-Non-mobilizable**	-0.732	1.175	-0.623	0.5382
**Serovar-Anatum**	-0.5467	3.961	-0.138	0.8912
**Serovar-Bovismorbificans**	-1.076	2.839	-0.379	0.7075
**Serovar-Braenderup**	-5.973	5.080	-1.176	0.2492
**Serovar-Choleraesuis**	-2.681	2.374	-1.129	0.2681
**Serovar-Enteritidis**	-0.4364	2.614	-0.167	0.8686
**Serovar-Give**	1.610	2.849	0.565	0.5764
**Serovar-I 4,[5],12:i:-**	$9.236x10^{-2}$	2.183	$4.2x10^{-2}$	0.9665
**Serovar-Indiana**	4.246	4.291	0.99	0.3306
**Serovar-Kedougou**	-5.888	5.128	-1.148	0.2602
**Serovar-London**	0.1783	2.698	$6.6x10^{-2}$	0.9478
**Serovar-Mbandaka**	0.6437	5.164	0.125	0.9017
**Serovar-Newport**	-0.7142	2.328	-0.307	0.7612
**Serovar-Ohio**	-0.3499	3.5820	$-9.8x10^{-2}$	0.9229

**Serovar-Panama**	-2.156	2.778	-0.776	0.4439
**Serovar-Rissen**	-0.1473	3.357	-4.4×10^{-2}	0.9653
**Serovar-Stanley**	-1.076	2.839	-0.379	0.7075
**Serovar-Typhimurium**	-0.3740	2.221	-0.168	0.8675
**Serovar-Weltevreden**	-21.54	8.615	-2.501	1.83×10^{-2}
**Source-Human**	0.2849	1.838	0.155	0.8779
**Infection-Gastrointestinal**	-9.956×10^{-2}	1.917	-5.2×10^{-2}	0.9589
**Infection-Invasive**	0.1103	1.977	5.6×10^{-2}	0.9559
**Phenotype-Rare**	-0.4611	0.5217	-0.884	0.3841
**IncQ1_1**	1.411	1.626	0.868	0.3925
**IncA_C2_1**	-8.376	5.995	-1.397	0.1729
**p0111_1**	-4.739	3.870	-1.225	0.2306
**IncFIB(K)_1_Kpn3**	-3.118	2.696	-1.157	0.2569
**IncFIA(HI1)_1_HI1**	-2.036	1.521	-1.339	0.191
**IncHI1A_1/IncHI1B(R27)_1_R27**	-8.714	3.865	-2.254	3.19×10^{-2}
**IncFII(S)_1**	4.715	1.754	2.689	1.18×10^{-2}
**IncHI2_1/IncHI2A_1**	-3.483	2.171	-1.604	0.1195
**IncFIB(S)_1**	-0.2413	0.9947	-0.243	0.81
**IncI1_1_Alpha**	-1.258	1.939	-0.649	0.5215
**IncI2_1**	-0.2682	2.425	-0.111	0.9127
**IncFII(p96A)_1_p96A**	NA	NA	NA	NA
**IncX1_4**	-1.257	1.885	-0.666	0.5108
**IncN_1**	0.2011	1.990	0.101	0.9202
**IncFIA_1/IncFII_1**	-1.582	2.887	-0.548	0.588
**IncQ2_1**	-1.632	1.988	-0.821	0.4185
**ColpVC_1**	-0.5261	3.008	-0.175	0.8624
**ISNCY**	0.5847	1.319	0.443	0.6608
**IS200_IS605**	5.736	3.746	1.531	0.1365
**IS6/IS26**	0.1354	0.1865	0.726	0.4737
**IS4**	-0.3703	0.7370	-0.502	0.6192
**IS1**	2.369	0.8209	2.886	7.3×10^{-3}
**IS256**	NA	NA	NA	NA
**IS3**	0.8052	0.6759	1.191	0.2432
**IS91**	6.602	5.659	1.167	0.2529
**IS1182**	-1.741	2.064	-0.844	0.4057
**IS630**	1.863	1.239	1.503	0.1435
**IS30**	NA	NA	NA	NA
**IS21**	-1.069	0.9083	-1.176	0.249
**IS5**	0.2407	1.842	0.131	0.897
**IS110**	0.2207	1.740	0.127	0.8999
**ISL3**	-5.755	6.515	-0.883	0.3843
**New**	-1.095	0.9566	-1.144	0.2619
**IS1380**	6.497×10^{-2}	1.794	3.6×10^{-2}	0.9714
**ISKRA4**	-1.091	2.887	-0.378	0.7083

**IS66**	-10.40	6.317	-1.647	0.1104

Statistic	Value
**Residual standard error**	1.402 on 29 degrees of freedom
**Multiple R-squared**	0.9168
**Adjusted R-squared**	0.7531
**F-statistic**	5.603 on 57 and 29 degrees of freedom
**p-value**	1.762×10^{-6}

Partial-F test mobility

Model	Residual degrees of freedom	Residual sum of squares	Degrees of freedom	Sum of squares	F-value	p-value
**Virulence plasmid**	29	57.04				
**Virulence plasmid minus mobility**	31	58.45	-2	-1.408	0.3579	0.7022

Partial-F test serovar

Model	Residual degrees of freedom	Residual sum of squares	Degrees of freedom	Sum of squares	F-value	p-value
**Virulence plasmid**	29	57.04				
**Virulence plasmid minus serovar**	45	90.95	-16	-33.91	1.078	0.4165

Partial-F test infection

Model	Residual degrees of freedom	Residual sum of squares	Degrees of freedom	Sum of squares	F-value	p-value
**Virulence plasmid**	29	57.04				
**Virulence plasmid minus infection**	47	92.47	-18	-35.43	1.001	0.4861

Partial-F test phenotype

Model	Residual degrees of freedom	Residual sum of squares	Degrees of freedom	Sum of squares	F-value	p-value
**Virulence plasmid**	29	57.04				
**Virulence plasmid minus phenotype**	32	60.33	-3	-3.290	0.5575	0.6473

Partial-F test source

Model	Residual degrees of freedom	Residual sum of squares	Degrees of freedom	Sum of squares	F-value	p-value
**Virulence plasmid**	29	57.04				
**Virulence plasmid minus source**	32	58.51	-3	-1.466	0.2484	0.9618

Partial-F test plasmid type

Model	Residual degrees of freedom	Residual sum of squares	Degrees of freedom	Sum of squares	F-value	p-value
**Virulence plasmid**	29	57.04				
**Virulence plasmid minus plasmid type**	44	195.5	-15	-138.4	4.692	1.82×10^{-4}

Metal-tolerance gene plasmid linear regression model

Linear regression formula:

Metal-tolerance ~ Factor(Mobility) + Factor(Serovar) + Factor(Source) + Factor(Infection) + Factor(Phenotype) + IncQ1_1 + IncA_C2_1 + p0111_1 + IncFIB(K)_1_Kpn3 + IncFIA(HI1)_1_HI1 + IncHI1A_1/IncHI1B(R27)_1_R27 + IncFII(S)_1 + IncHI2_1/IncHI2A_1 + IncFIB(S)_1 + IncI1_1_Alpha + IncI2_1 + IncFII(p96A)_1_p96A + IncX1_4 + IncN_1 + IncFIA_1_IncFII_1 + IncQ2_1 + ColpVC_1 + ISNCY + IS200_IS605 + IS6/IS26 + IS4 + IS1 + IS256 + IS3 + IS91 + IS1182 + IS630 + IS30 + IS21 + IS5 + IS110 + ISL3 + new + IS1380 + ISKRA4 + IS66

Residuals:

Minimum	First quartile	Median	Third quartile	Maximum
-2.993	-0.3158	0.0000	0.3136	3.952

Coefficient	Estimate	Standard error	t-value	p-value
**Intercept**	1.200	2.665	0.45	0.6557
**Mobility-Mobilizable**	-0.3687	1.164	-0.317	0.7537
**Mobility-Non-mobilizable**	-0.8073	1.309	-0.617	0.5420
**Serovar-Anatum**	-0.4819	4.410	-0.109	0.9138
**Serovar-Bovismorbificans**	-1.678	3.161	-0.531	0.5997
**Serovar-Braenderup**	2.910	5.657	0.514	0.6108
**Serovar-Choleraesuis**	-4.017	2.644	-1.52	0.1395
**Serovar-Enteritidis**	-2.554	2.911	-0.877	0.3876
**Serovar-Give**	-0.9556	3.1727	-0.301	0.7654
**Serovar-I 4,[5],12:i:-**	-0.1029	2.431	$-4.2x10^{-2}$	0.9665
**Serovar-Indiana**	1.290	4.778	0.27	0.7891
**Serovar-Kedougou**	8.466	5.710	1.483	0.1489
**Serovar-London**	-2.944	3.004	-0.98	0.3351
**Serovar-Mbandaka**	-3.599	5.750	-0.626	0.5364
**Serovar-Newport**	-1.762	2.592	-0.68	0.5020
**Serovar-Ohio**	-0.7182	3.989	-0.18	0.8584
**Serovar-Panama**	-1.083	3.093	-0.35	0.7288
**Serovar-Rissen**	-0.1182	3.738	$-3.2x10^{-2}$	0.975
**Serovar-Stanley**	-1.6778	3.1613	-0.531	0.59965
**Serovar-Typhimurium**	-1.9125	2.4733	-0.773	0.44562
**Serovar-Weltevreden**	-11.79	9.5924	-1.229	0.22893
**Source-Human**	0.6963	2.046	0.34	0.7361
**Infection-Gastrointestinal**	-0.4556	2.134	-0.213	0.8325
**Infection-Invasive**	-1.156	2.202	-0.525	0.6037
**Phenotype-Rare**	-0.3711	0.5809	-0.639	0.5280
**IncQ1_1**	4.904	1.811	2.708	$1.122x10^{-2}$
**IncA_C2_1**	5.873	6.676	0.88	0.3863
**p0111_1**	-1.268	4.310	-0.294	0.7708
**IncFIB(K)_1_Kpn3**	0.2166	3.002	$7.2x10^{-2}$	0.9430
**IncFIA(HI1)_1_HI1**	5.055	1.694	2.985	$5.71x10^{-3}$
**IncHI1A_1/IncHI1B(R27)_1_R27**	-5.821	4.304	-1.352	0.1867
**IncFII(S)_1**	0.5173	1.953	0.265	0.7930
**IncHI2_1/IncHI2A_1**	7.143	2.418	2.955	$6.16x10^{-3}$
**IncFIB(S)_1**	0.1527	1.108	0.138	0.8913
**IncI1_1_Alpha**	-1.200	2.160	-0.556	0.5825
**IncI2_1**	0.6923	2.700	0.256	0.7994
**IncFII(p96A)_1_p96A**	NA	NA	NA	NA
**IncX1_4**	1.968	2.099	0.938	0.3561
**IncN_1**	4.662	2.216	2.104	$4.419x10^{-2}$

**IncFIA_1/IncFII_1**	-1.892	3.215	-0.588	0.5608
**IncQ2_1**	-0.849	2.214	-0.384	0.7041
**ColpVC_1**	-0.393	3.349	-0.117	0.9074
**ISNCY**	1.163	1.469	0.792	0.4349
**IS200_IS605**	6.832	4.171	1.638	0.1122
**IS6/IS26**	-0.1008	0.2077	-0.485	0.6310
**IS4**	1.007	0.8207	1.227	0.2297
**IS1**	-0.3818	0.9141	-0.418	0.6792
**IS256**	NA	NA	NA	NA
**IS3**	-0.9287	0.7526	-1.234	0.2271
**IS91**	-6.294	6.301	-0.999	0.3261
**IS1182**	6.9167	2.2973	3.011	5.35×10^{-3}
**IS630**	1.122	1.380	0.813	0.4229
**IS30**	NA	NA	NA	NA
**IS21**	1.507	1.011	1.49	0.1470
**IS5**	-0.6058	2.052	-0.295	0.7699
**IS110**	-0.9661	1.937	-0.499	0.6217
**ISL3**	23.11	7.254	3.186	3.44×10^{-3}
**New**	0.3975	1.065	0.373	0.7118
**IS1380**	0.88	1.998	0.44	0.6629
**ISKRA4**	-1.301	3.215	-0.405	0.6888
**IS66**	4.113	7.034	0.585	0.5633

**Statistic**	**Value**
**Residual standard error**	1.562 on 29 degrees of freedom
**Multiple R-squared**	0.9414
**Adjusted R-squared**	0.8262
**F-statistic**	8.172 on 57 and 29 degrees of freedom
**p-value**	2.13×10^{-8}

Partial-F test mobility

**Model**	**Residual degrees of freedom**	**Residual sum of squares**	**Degrees of freedom**	**Sum of squares**	**F-value**	**p-value**
**Virulence plasmid**	29	70.73				
**Virulence plasmid minus mobility**	31	71.66	-2	-0.9287	0.1904	0.8277

Partial-F test serovar

Model	Residual degrees of freedom	Residual sum of squares	Degrees of freedom	Sum of squares	F-value	p-value
**Virulence plasmid**	29	70.73				
**Virulence plasmid minus serovar**	45	112.2	-16	-41.51	1.064	0.4279

Partial-F test infection

Model	Residual degrees of freedom	Residual sum of squares	Degrees of freedom	Sum of squares	F-value	p-value
**Virulence plasmid**	29	70.73				
**Virulence plasmid minus infection**	47	124.6	-18	-53.87	1.227	0.3037

Partial-F test phenotype

Model	Residual degrees of freedom	Residual sum of squares	Degrees of freedom	Sum of squares	F-value	p-value
**Virulence plasmid**	29	70.73				
**Virulence plasmid minus phenotype**	32	73.11	-3	-2.379	0.3251	0.8071

Partial-F test source

Model	Residual degrees of freedom	Residual sum of squares	Degrees of freedom	Sum of squares	F-value	p-value
**Virulence plasmid**	29	70.73				
**Virulence plasmid minus source**	32	71.91	-3	-1.182	0.1615	0.9214

Partial-F test plasmid type

**Model**	**Residual degrees of freedom**	**Residual sum of squares**	**Degrees of freedom**	**Sum of squares**	**F-value**	**p-value**
**Virulence plasmid**	29	70.73				
**Virulence plasmid minus source**	44	206.1	-15	-135.4	3.701	1.233×10^{-3}

Appendix F. Chromosome arrangements

Of the 68 assemblies that passed quality control, 60 had the common chromosome arrangement GS1.0, three had the less common chromosome arrangements GS0.48, GS1.123, GS0.121, and five had fragmented chromosomes that met the rest of the QC criteria (Fig S21).

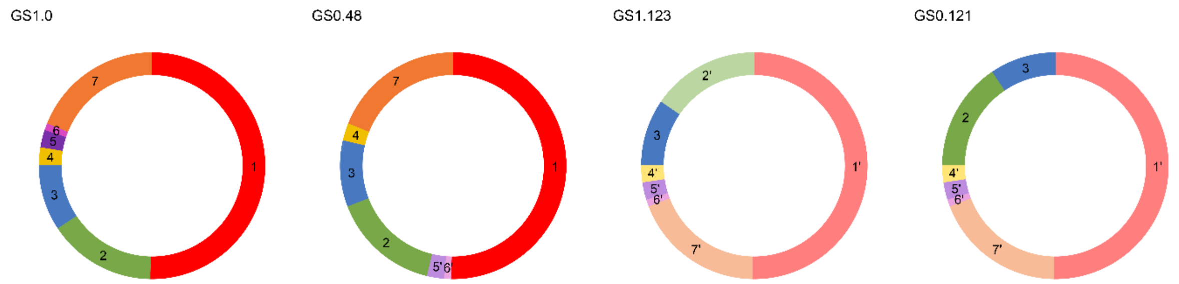

Fig S21. Chromosomal arrangements of non-typhoidal *Salmonella* examined in this study.

Appendix G. Genome assembly comparison

To determine which assembler gave the best assemblies for the *Salmonella* isolates, four methods were trailed on five sets of PacBio and Illumina reads:

1. Canu-pilon – The PacBio reads were trimmed and assembled using Canu v1.8 [1], before the Burrows-Wheeler Aligner (BWA) v0.7.17 [2] was used to align Illumina reads to the assembly and Pilon v1.22 [3] was used to correct mismatches. Five rounds of BWA and Pilon were performed.
2. Flye-pilon – The PacBio reads were trimmed and assembled using Flye v2.7 [4], before five rounds of BWA with Illumina reads and Pilon.
3. Spades-hybrid-pilon – The PacBio reads were trimmed and assembled with Illumina reads using hybridSpades v3.13.1 [5], before five rounds of BWA with Illumina reads and Pilon.
4. Unicycler-pilon – The PacBio reads were trimmed and assembled with Illumina reads using Unicycler v0.4.8 [6], before five rounds of BWA with Illumina reads and Pilon.

The Illumina and PacBio reads were aligned to the assemblies produced by the four methods using BWA. Socru v2.2.3 [7] was used to determine the chromosome arrangement of the assemblies. The assemblies were compared based on: contig sizes (Figure S22), mean Illumina and PacBio read depth (Figure S23 and S24), the number of linear and circulated contigs, the number of variants called with Illumina reads (Table S2), and the chromosome arrangements (Table S3).

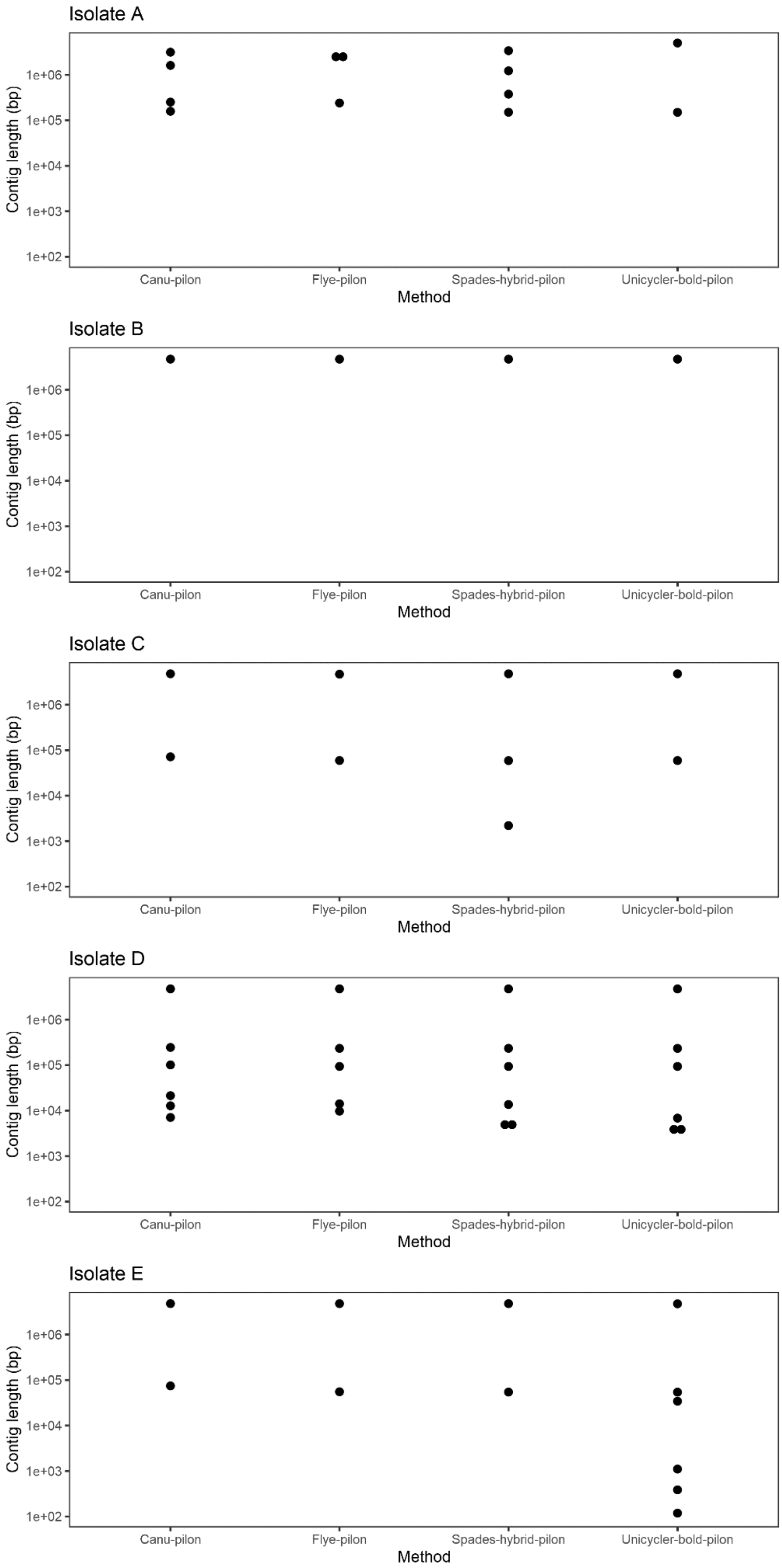

Fig S22. Dot plots of contig size versus assembly method for five *Salmonella* isolates.

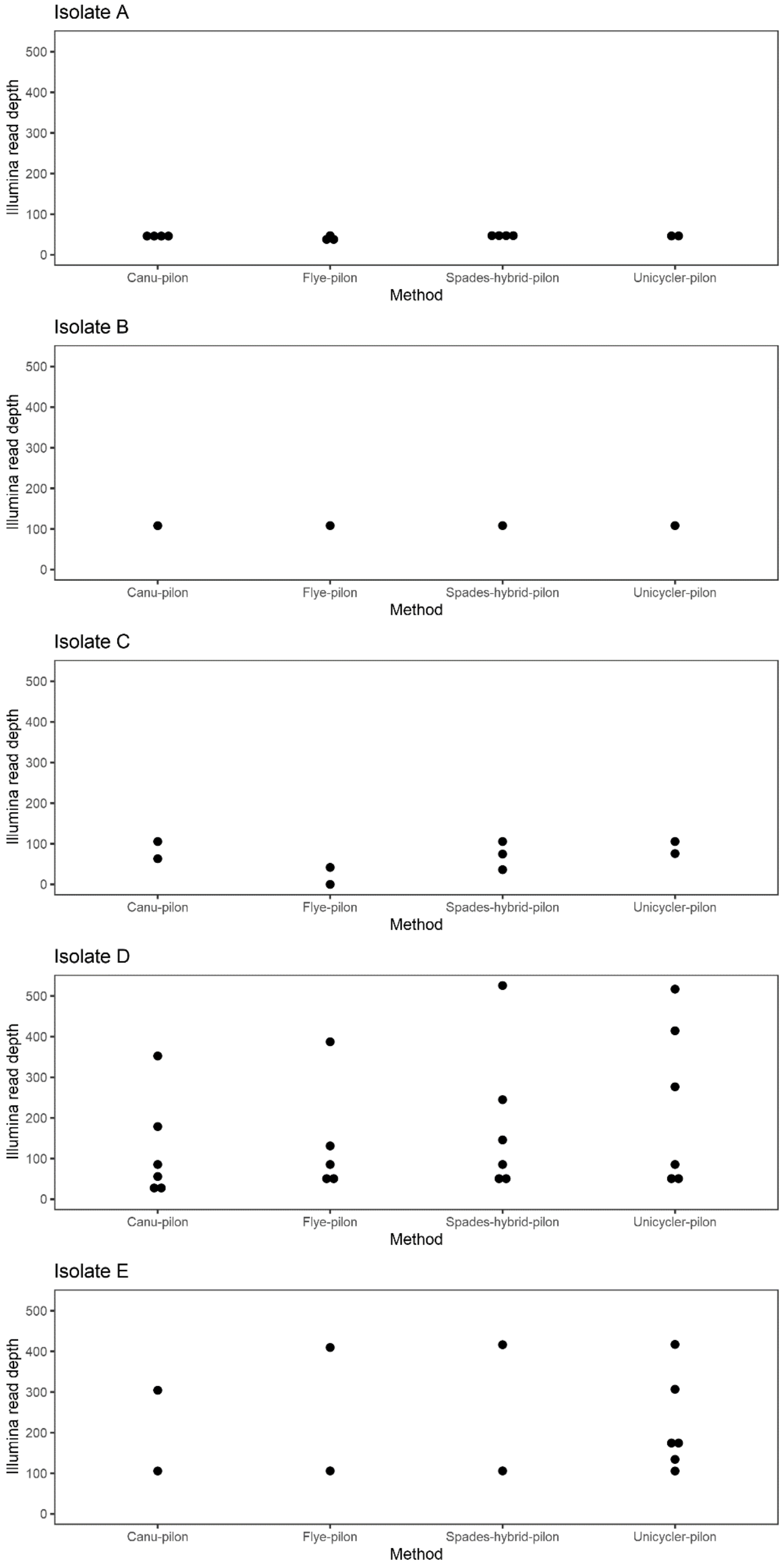

Fig S23. Dot plots of mean Illumina read depth for contigs versus assembly method for five *Salmonella* isolates.

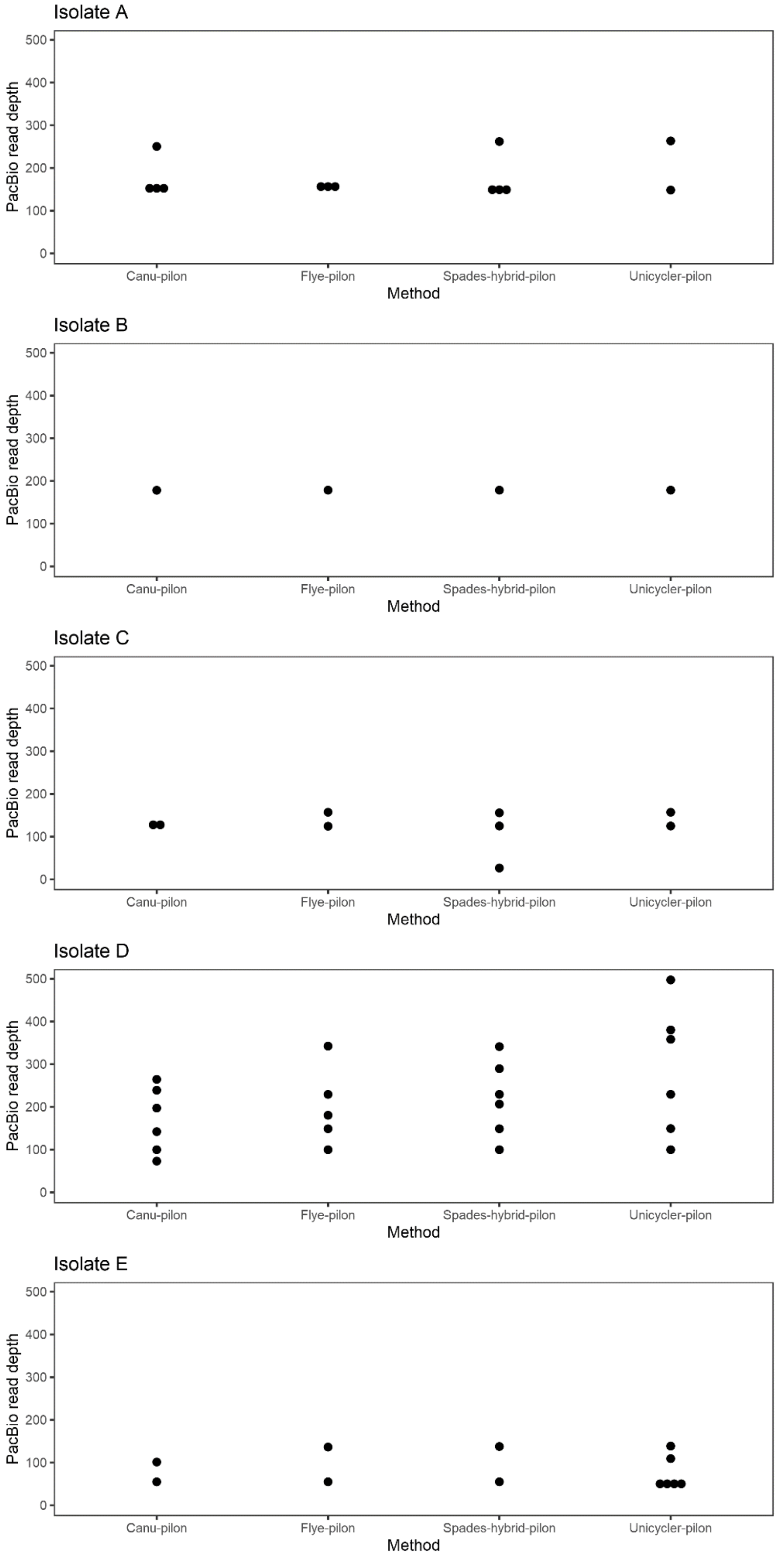

Fig S24. Dot plots of mean PacBio read depth for contigs versus assembly method for five *Salmonella* isolates.

Table S2. Contig types and variants with Illumina reads for five isolates assembled using different methods

Isolate	Method	Contigs		Illumina read variants
		Linear	Circular	
A	Flye-pilon	3	0	11
B	Flye-pilon	0	1	0
C	Flye-pilon	0	2	100
D	Flye-pilon	1	4	5
E	Flye-pilon	0	2	0
A	Canu-pilon	4	0	0
B	Canu-pilon	1	0	0
C	Canu-pilon	2	0	0
D	Canu-pilon	6	0	1
E	Canu-pilon	2	0	0
A	Spades-hybrid-pilon	4	0	3
B	Spades-hybrid-pilon	1	0	1
C	Spades-hybrid-pilon	2	1	1
D	Spades-hybrid-pilon	5	1	2
E	Spades-hybrid-pilon	2	0	5
A	Unicycler-pilon	0	2	2
B	Unicycler-pilon	0	1	0
C	Unicycler-pilon	0	2	0
D	Unicycler-pilon	0	6	0
E	Unicycler-pilon	5	1	10

Table S3. Socru outputs for five isolates assembled using different methods

Isolate	Method	Arrangement	Fragment positions A	B	C	D	E	F	G
A	Canu-pilon	GS0.2	1	?	2'				
A	Flye-pilon	GS0.0	7	1	5	?	?		
A	Spades-hybrid-pilon	GS0.2	1'	?	1'				
A	Unicycler-bold-pilon	GS1.0	1	2	3	4	5	6	7
B	Canu-pilon	GS1.0	1	2	3	4	5	6	7
B	Flye-pilon	GS1.0	1	2	3	4	5	6	7
B	Spades-hybrid-pilon	GS1.0	1	2	3	4	5	6	7
B	Unicycler-bold-pilon	GS1.0	1	2	3	4	5	6	7
C	Canu-pilon	GS0.121	1'	7'	6'	5'	4'	2	3
C	Flye-pilon	GS0.121	1'	7'	6'	5'	4'	2	3
C	Spades-hybrid-pilon	GS0.121	1'	7'	6'	5'	4'	2	3
C	Unicycler-bold-pilon	GS0.121	1'	7'	6'	5'	4'	2	3
D	Canu-pilon	GS1.0	1	2	3	4	5	6	7
D	Flye-pilon	GS1.0	1	2	3	4	5	6	7
D	Spades-hybrid-pilon	GS1.0	1	2	3	4	5	6	7
D	Unicycler-bold-pilon	GS1.0	1	2	3	4	5	6	7
E	Canu-pilon	GS1.0	1	2	3	4	5	6	7
E	Flye-pilon	GS1.0	1	2	3	4	5	6	7
E	Spades-hybrid-pilon	GS1.0	1	2	3	4	5	6	7
E	Unicycler-bold-pilon	GS1.0	1	2	3	4	5	6	7

The assemblies produced by the four methods varied in quality. The assemblies produced by Flye-pilon or Unicycler-pilon contained the most circulated contigs. The number of variants produced varied for each assembly, as did the Illumina and PacBio read depth. The Socru outputs were identical amongst the assembly methods, apart from Isolate A, where only Unicycler produced a chromosome that did not have duplicate or unknown fragments. Based on these results, it was decided that Flye-pilon and Unicycler-pilon would be used to assemble the reads, and the assembly chosen would be based on Socru, the number of

Illumina variants and the number of completed contigs, along with the presence of AMR genes and plasmid types.

Appendix G. Pseudogene analysis

Pseudogenes were identified using the database and method described by Mather *et al*. [8]. The database used was formed from the coding sequences of the *S. enterica* serovar Typhimurium strain SL1344 chromosome (accession FQ312003). A linear regression model was used to model the total number of pseudogenes with the isolate metadata in R v3.6.1 [9]. The genetic distance of the assemblies to the reference genome as calculated by Mash v1.1 [10] and genome size of assemblies as calculated by QUAST v5.0.2 [11] were also included as explanatory variables. Partial-F tests were used to determine if any of the potential explanatory variables significantly improved the fit of the model.

Regression analysis demonstrated that *Salmonella* serovar was significantly associated with number of pseudogenes ($p < 2.2$ x 10-16). To determine if this result was confounded by the choice of comparator (*S*. Typhimurium strain SL1344), we used Mash to measure the genetic distance between the genome from which the database was created and each isolate along with the size of each assembly, as genome size may be associated with pseudogene acquisition. We found that genetic distance ($p=0.1217$) and genome size ($p=0.9132$) were not associated with the number of pseudogenes identified (Figure S5). Notably, *S*. Choleraesuis had a larger number of pseudogenes, and the *S*. Typhimurium and I 4,[5],12:i:- contained fewer pseudogenes in comparison to the other NTS serovars investigated (Figure S25), similar to previous studies [12]. Model fit was relatively poor due in large to the number of pseudogenes being trimodal.

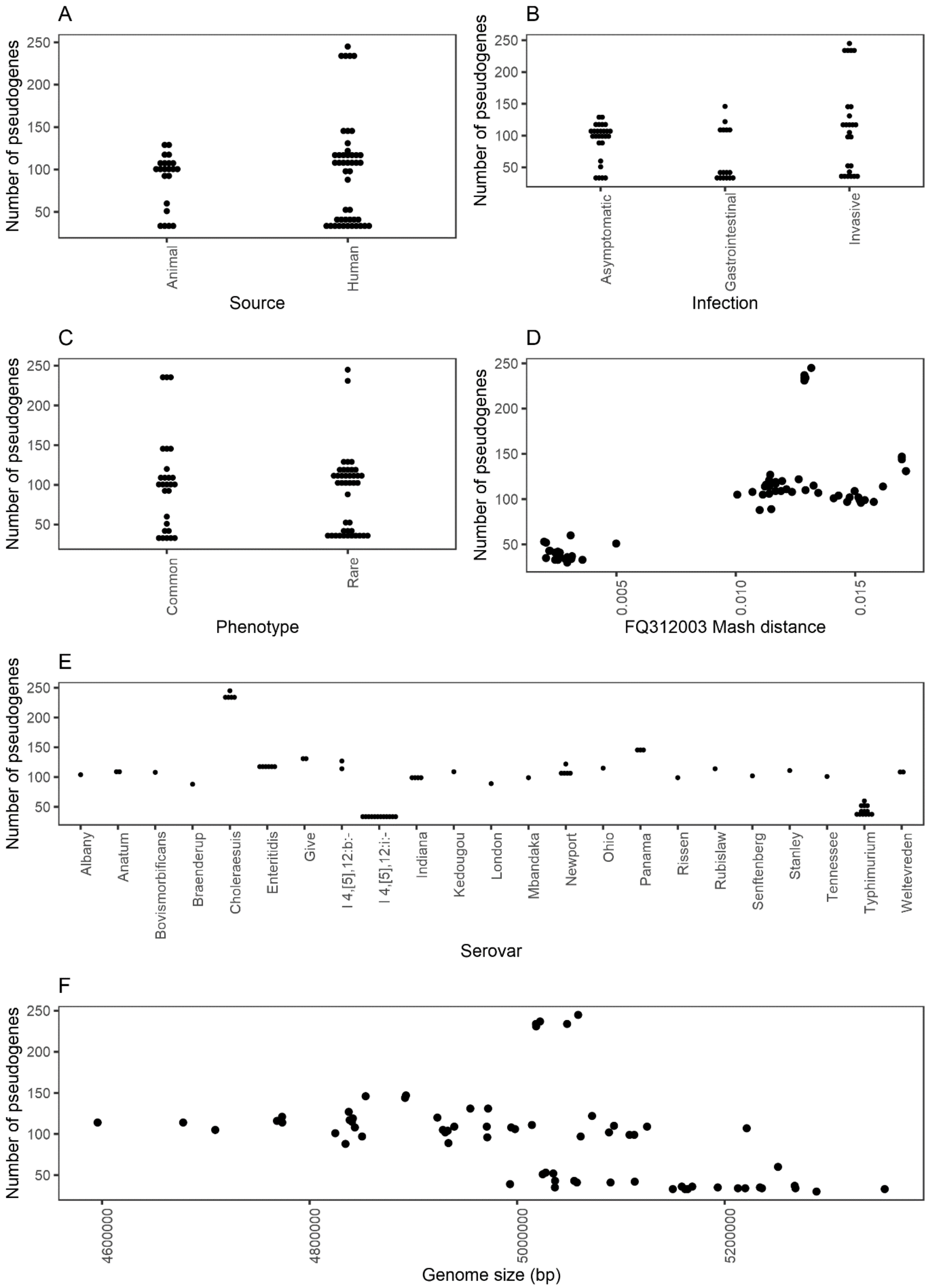

Fig S25. Dot plots of the number of pseudogenes versus source (A), infection type (B), AMR phenotype commonality (C) and serovar (D), along with a scatterplot of the number of pseudogenes versus the Mash distance between NTS isolates and the FQ312003 reference genome (E) and the genome size (F).

Pseudogene linear regression model

Linear regression formula:
Pseudogenes ~ FQ312003 Mash distance + Genome size + Factor(Serovar) + Factor(Source) + Factor(Infection) + Factor(Phenotype)

Residuals:

Minimum	First quartile	Median	Third quartile	Maximum
-10.67	-1.688	0	1.087	13.53

Coefficient	Estimate	Standard error	t-value	p-value
**Intercept**	67.63	49.45	1.368	0.1792
**FQ312003 Mash distance**	2,903	1,835	1.582	0.1217
**Genome size**	$-1.044\text{x}10^{-6}$	$1.201\text{x}10^{-5}$	$-8.7\text{x}10^{-2}$	0.9312
**Serovar-Anatum**	13.58	7.954	1.707	$9.58\text{x}10^{-2}$
**Serovar-Bovismorbificans**	12.03	8.599	1.400	0.1696
**Serovar-Braenderup**	-0.8982	10.87	-0.083	0.9346
**Serovar-Choleraesuis**	137.5	6.797	20.24	$<2\text{x}10^{-16}$
**Serovar-Enteritidis**	22.79	7.130	3.196	$2.758\text{x}10^{-3}$
**Serovar-Give**	19.53	8.797	2.220	$3.231\text{x}10^{-2}$
**Serovar-I 4,[5],12:b:-**	25.02	7.863	3.182	$2.872\ \text{x}10^{-3}$
**Serovar-I 4,[5],12:i:-**	-35.30	23.31	-1.514	0.1380
**Serovar-Indiana**	-7.999	6.146	-1.301	0.2008
**Serovar-Kedougou**	8.792	9.607	0.915	0.3657
**Serovar-London**	-6.824	9.088	-0.751	0.4572
**Serovar-Mbandaka**	-7.927	8.014	-0.989	0.3287
**Serovar-Newport**	16.12	7.905	2.039	$4.825\text{x}10^{-2}$
**Serovar-Ohio**	19.56	9.905	1.975	$5.543\text{x}10^{-2}$
**Serovar-Panama**	35.52	8.491	4.183	$1.58\text{x}10^{-4}$
**Serovar-Rissen**	-6.140	7.992	-0.768	0.4469
**Serovar-Rubislaw**	9.865	12.53	0.788	0.4357
**Serovar-Senftenberg**	-4.252	7.641	-0.556	0.5811
**Serovar-Stanley**	15.73	8.862	1.775	$8.376\text{x}10^{-2}$
**Serovar-Tennessee**	-2.451	7.778	-0.315	0.7544
**Serovar-Typhimurium**	-24.63	22.27	-1.086	0.2841

**Serovar-Weltevreden**	9.167	7.651	1.198	0.2381
**Source-Human**	-5.546	6.099	-0.909	0.3688
**Infection-Gastrointestinal**	3.235	6.136	0.527	0.6010
**Infection-Invasive**	4.301	6.176	0.696	0.4903
**Phenotype-Rare**	-7.283×10^{-2}	1.839	-4×10^{-2}	0.9686

**Statistic**	**Value**
**Residual standard error**	5.284 on 39 degrees of freedom
**Multiple R-squared**	0.9945
**Adjusted R-squared**	0.9905
**F-statistic**	251.7 on 28 and 39 degrees of freedom
**p-value**	$<2.2 \times 10^{-16}$

Partial-F test serovar

**Model**	**Residual degrees of freedom**	**Residual sum of squares**	**Degrees of freedom**	**Sum of squares**	**F-value**	**p-value**
**Pseudogene**	39	1,089				
**Pseudogene minus serovar**	61	70,621	-22	-69,553	113.2	$<2.2 \times 10^{-16}$

Partial-F test source

**Model**	**Residual degrees of freedom**	**Residual sum of squares**	**Degrees of freedom**	**Sum of squares**	**F-value**	**p-value**
**Pseudogene**	39	1,088.9				
**Pseudogene minus source**	40	1,112	-1	-23.09	0.8269	0.3688

Partial-F test Infection

**Model**	**Residual degrees of freedom**	**Residual sum of squares**	**Degrees of freedom**	**Sum of squares**	**F-value**	**p-value**
**Pseudogene**	39	1,088.9				
**Pseudogene minus infection**	41	1.106.7	-2	-17.76	0.318	0.7295

Partial-F test source

Model	Residual degrees of freedom	Residual sum of squares	Degrees of freedom	Sum of squares	F-value	p-value
**Pseudogene**	39	1,088.9				
**Pseudogene minus infection**	40	1,089	-1	-0.0438	0.0016	0.9686

References

1. **Koren S, Walenz BP, Berlin K, Miller JR, Bergman NH, *et al.*** Canu: scalable and accurate long-read assembly via adaptive k-mer weighting and repeat separation. *Genome Res* 2017;27:722–736.

2. **Li H, Durbin R**. Fast and accurate short read alignment with Burrows-Wheeler transform. *Bioinformatics* 2009;25:1754–1760.

3. **Walker BJ, Abeel T, Shea T, Priest M, Abouelliel A, *et al.*** Pilon: an integrated tool for comprehensive microbial variant detection and genome assembly improvement. *PLoS One* 2014;9:1–14.

4. **Kolmogorov M, Yuan J, Lin Y, Pevzner PA**. Assembly of long, error-prone reads using repeat graphs. *Nat Biotechnol* 2019;37:540–546.

5. **Antipov D, Korobeynikov A, McLean JS, Pevzner PA**. HybridSPAdes: An algorithm for hybrid assembly of short and long reads. *Bioinformatics* 2016;32:1009–1015.

6. **Wick RR, Judd LM, Gorrie CL, Holt KE**. Unicycler: Resolving bacterial genome assemblies from short and long sequencing reads. *PLOS Comput Biol* 2017;13:1–22.

7. **Page AJ, Ainsworth E V, Langridge GC**. socru: typing of genome-level order and orientation around ribosomal operons in bacteria. *Microb Genomics* 2020;6:1–6.

8. **Mather AE, Phuong TLT, Gao Y, Clare S, Mukhopadhyay S, *et al.*** New variant of multidrug-resistant *Salmonella enterica* serovar Typhimurium associated with invasive disease in immunocompromised patients in Vietnam. *MBio* 2018;9:1–11.

9. **R core team**. R: a language and environment for statistical computing. 2019;1–1.

10. **Ondov BD, Starrett GJ, Sappington A, Kostic A, Koren S, *et al.*** Mash Screen: high-throughput sequence containment estimation for genome discovery. *Genome Biol* 2019;20:1–13.

11. **Gurevich A, Saveliev V, Vyahhi N, Tesler G**. QUAST: quality assessment tool for genome assemblies. *Bioinformatics* 2013;29:1072–1075.

12. **Fricke WF, Mammel MK, McDermott PF, Tartera C, White DG, *et al.*** Comparative genomics of 28 *Salmonella enterica* isolates: Evidence for CRISPR-mediated adaptive sublineage evolution. *J Bacteriol* 2011;193:3556–3568.
